# Physiological and biochemical responses of ‘Divadona’ peach on Rootpac 20 and Rootpac 40 under drought and heat stress adaptation and its recovery mechanisms

**DOI:** 10.1111/ppl.70250

**Published:** 2025-05-07

**Authors:** Meral Dogan, Ibrahim Bolat, Metin Turan, Ozkan Kaya

**Affiliations:** ^1^ Department of Horticulture Harran University, Graduate School of Natural and Applied Sciences Sanliurfa Turkey; ^2^ Department of Horticulture Harran University, Faculty of Agriculture Sanliurfa Turkey; ^3^ Faculty of Economy and Administrative Science Yeditepe University Istanbul Turkey; ^4^ Department of Life Sciences Western Caspian University Baku Azerbaijan; ^5^ Republic of Turkey Ministry of Agriculture and Forestry Erzincan Horticultural Research Institute Erzincan Turkey

## Abstract

Climate change‐induced drought and heat stress pose significant challenges to global peach production, threatening agricultural sustainability and food security. This study, therefore, investigated the morphological, physiological and biochemical responses of the ‘Divadona'peach cultivar grafted onto two different rootstocks (Rootpac 20 and Rootpac 40) under drought stress, heat shock, and their combination. We aimed to identify superior rootstock performances and understand stress tolerance mechanisms for improved cultivation strategies. Our findings revealed that combined stress induced the most severe impacts, with Rootpac 40 demonstrating superior stress tolerance. Under combined stresses, relative shoot diameter decreased less in Rootpac 40/‘Divadona’ (19.75%) compared to Rootpac 20/‘Divadona’, while relative shoot length showed similar patterns. Antioxidant enzyme activities increased significantly, with POD showing the highest elevation in Rootpac 40/‘Divadona’ compared to Rootpac 20/‘Divadona. Stress markers exhibited substantial accumulation, with MDA content rising more in Rootpac 20/‘Divadona’ than in Rootpac 40/‘Divadona’. Nutrient analysis showed that Rootpac 40/‘Divadona’ maintained higher levels of essential nutrients under stress, with nitrogen content declining less compared to Rootpac 20/‘Divadona’. The study demonstrated that Rootpac 40/‘Divadona’ possesses superior stress tolerance mechanisms through better maintenance of growth parameters, enhanced antioxidant defense systems, and improved nutrient retention capacity. These findings provide valuable insights for fruit growing, enabling informed rootstock selection for peach cultivation in drought‐prone regions, ultimately contributing to more resilient and sustainable fruit production systems under changing climatic conditions.

## 1 INTRODUCTION

Abiotic stress factors caused by global climate change create significant constraints in agricultural production and pose serious challenges in the sustainable cultivation of perennial fruit trees (Zandalinas et al., 2018; Kaya et al., 2021; Kaya and Kose, 2022). Among these stress factors, drought, high temperature, late spring and early frosts, and winter cold are important abiotic stressors that frequently occur simultaneously and lead to complex changes at physiological, biochemical, and molecular levels in plants (Suzuki et al., 2014; Dogan et al., 2022; Bakir et al., 2022). The synergistic effect of these stress factors significantly influences plants' survival strategies and determines the efficacy of their adaptation mechanisms (Bolat et al., 2022,2024). In this context, grafting techniques and rootstock selection, which are widely used in commercial cultivation of *Prunus* species, are critical for developing abiotic stress tolerance (Dogan et al., 2025). The Rootpac series rootstocks in particular stand out among newly developed generation rootstocks in terms of stress tolerance (Jiménez et al., 2013). Among these rootstocks, Rootpac 20 and Rootpac 40 hold special significance due to their adaptation capabilities to different stress conditions and compatibility with grafted varieties (Dogan et al., 2025). The stress tolerance mechanisms of these two important rootstocks from the Rootpac series form a complex network on cellular and molecular levels (Gainza et al., 2015). These defense systems encompass interrelated processes such as regulation of stomatal conductance, accumulation of osmolytes, activation of antioxidant systems, and maintenance of mineral matter homeostasis (Wahid et al., 2007; Dogan et al., 2025). Notably, Rootpac 40 rootstock's superior performance in coordinating these mechanisms indicates its remarkable adaptation capability under stress conditions (Opazo et al., 2020).

Plants possess various adaptation mechanisms they have developed against drought and high‐temperature stress. However, the accumulation of reactive oxygen species (ROS) in response to stress conditions in plants and the oxidative damage they cause are important factors affecting plants' stress tolerance (Kaya, 2020; Seyed Hajizadeh et al., 2023). Cellular protection mechanisms such as activation of antioxidant defense systems and osmolyte accumulation play critical roles in limiting this damage and maintaining cellular homeostasis (Noctor et al., 2014). In this context, the antioxidant capacity and osmoregulation abilities exhibited by different rootstocks under stress conditions are considered important indicators in determining their stress tolerance (Szabados and Savouré, 2010). On the other hand, mineral nutrition and water relations are other factors that play important roles in regulating stress tolerance. Changes in mineral uptake, transport, and utilization efficiency under stress conditions directly affect plants' adaptation capacity (Ahanger et al., 2016). In particular, balanced uptake and utilization of macro and micronutrients are critically important in enhancing stress tolerance and optimizing post‐stress recovery processes (Rahman et al., 2020). In this context, research on the stress tolerance of rootstock‐scion combinations in stone fruit species is of great importance for understanding the adaptation mechanisms developed by different genotypes against stress and developing resistant combinations that can cope with future challenges of climate change (Jiménez et al., 2013; Kaya et al., 2020). Within this scope, elucidating the molecular and physiological bases of rootstock‐scion interactions provides critical information for sustainable fruit cultivation.

The ‘Divadona’ peach cultivar is characterized by vigorous growth and a semi‐open canopy structure. It exhibits early‐season blooming with abundant flowering, leading to consistently high annual fruit production (Anonymous, 2025). However, information on the various morphological, physiological, and biochemical parameters of ‘Divadona'in response to drought and heat shock adaptation, as well as subsequent recovery, remains limited. This knowledge gap is particularly evident when considering its performance on Rootpac 20 and Rootpac 40 rootstocks. Understanding these responses is crucial for optimizing stress resilience and improving overall tree productivity. When examining the current literature and considering the physiological and biochemical aspects of stress responses, the following research objective emerges was to elucidate the stress adaptation mechanisms and recovery responses of the ‘Divadona'peach cultivar grafted onto Rootpac 20 and Rootpac 40 rootstocks under combined drought and heat stress conditions. Through comprehensive analysis of some morphological parameters, physiological indicators, and biochemical markers, this research sought to illuminate the complex stress response networks activated in these rootstock‐scion combinations. The investigation of these parameters during both stress exposure and recovery periods provides valuable insights into the differential adaptation strategies employed by these specific rootstock‐scion combinations, contributing to our understanding of stress tolerance mechanisms in *Prunus* species and supporting the development of climate‐resilient cultivation practices in commercial peach production.

## 2 MATERIALS AND METHODS

### 2.1 Plant growth conditions and treatments

The study utilized one‐year‐old ‘Divadona’ peach cultivars grafted onto Rootpac 20 and Rootpac 40 rootstocks which were propagated through tissue culture and obtained from a commercial company (Agromillora and Elma Tarim). Saplings were cultivated in a glasshouse located at the Faculty of Agriculture, Harran University (38°59′ L.E, 37°10′ L.N, 530 m a.s.l.). The greenhouse maintained a controlled environment with a daytime temperature of 25 ± 2°C and a nighttime temperature of 21 ± 2°C. The experiment was conducted under glasshouse conditions with Photosynthetically Active Radiation (PAR) levels ranging from 650 to 840 μmol m^2^ s^−1^ and relative humidity controlled at 50 ± 5%. The grafted plants were planted in mid‐March 2022. The grafted saplings used in the experiment were not treated with any plant growth regulators during planting. The development of the saplings occurred entirely under natural conditions. Uniformly developing plants were selected and planted in 12 L plastic containers filled with equal amounts of peat. Klasmann peat was used as the growing medium, with specifications of EC:35 mS/m, pH:6.5, and fertilizer content of 14:10:18 (N:P:K) at 1.0 kg m^3‐1^ (Klasmann, TS1, Klasmann‐Deilman Gmbh). The pruning process was performed based on the overall height and structure of the plants, ensuring uniformity among all saplings. The saplings were pruned to a height of approximately 25–30 cm, leaving 3–4 buds on the shoots. The planted cultivars were allowed to develop for approximately eight weeks during the spring period. The applications in the experiment began on May 9, 2022. Until the beginning of the experiment, all plants were irrigated equally at 100% pot capacity, and this moisture level was maintained, and also 50% Hoagland solution was manually applied every seven days for plant growth. To prevent any potential positional effects, the containers were randomly repositioned within the greenhouse on a weekly basis. The experiment was established in a randomized complete block design with three replications and five plants per replication in a factorial arrangement. The study comprised 120 plants in total, including four treatment combinations (Control, Drought, Heat Shock, and Drought + Heat Shock) and the ‘Divadona’ peach cultivar grafted onto two different rootstocks (Rootpac 40 and Rootpac 20).

**FIGURE 1:**
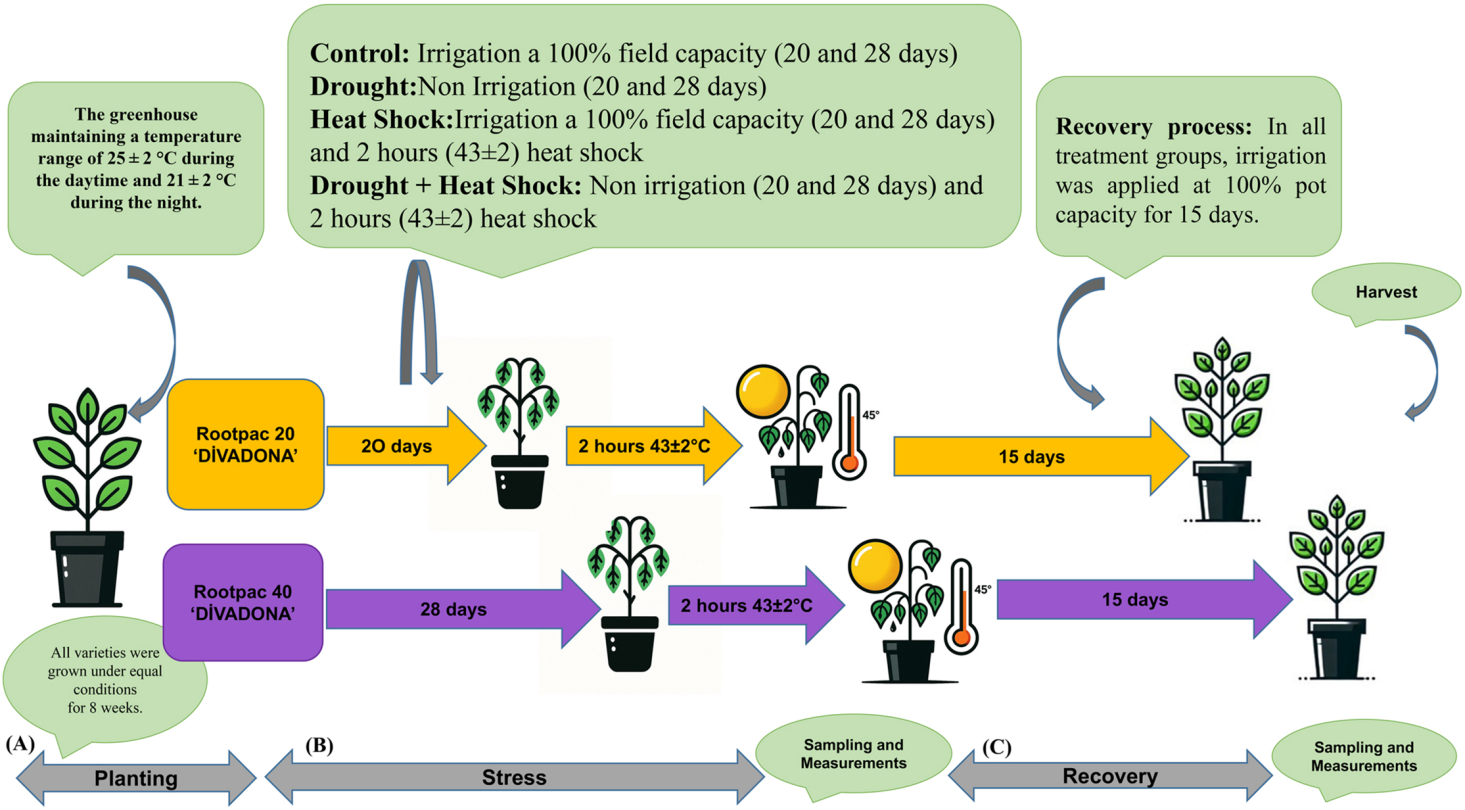
Experimental design for assessing the impact of drought and heat shock on plant growth and the recovery process under controlled greenhouse conditions.

Drought stress in grafted plants was monitored through leaf wilting observation, while control and heat shock combinations were irrigated at predetermined pot capacity. Irrigation was withheld in drought and heat shock + drought combinations until visible leaf wilting occurred. The number of days until leaf wilting varied among the grafted plants. Accordingly, stress treatments commenced on May 9, 2022, for all plant groups. Control and heat shock groups were weighed and irrigated to pot capacity every two days, while drought and drought+heat shock groups received no irrigation. On the 20th day of stress treatment (May 29, 2022), leaf wilting was first observed in ‘Divadona’ peach samplings grafted onto Rootpac 20 rootstock. The saplings grafted onto Rootpac 40 rootstock began wilting on the 28^th^ day (June 6, 2022). Subsequently, the high temperature and drought+heat shock groups were exposed to heat shock (43 ± 2°C) for 2 h, completing the stress phase (Figure 1). Upon completion of the stress phase, physiological measurements were taken for all treatment combinations, and samples were collected in liquid nitrogen for biochemical analyses and stored at −80°C. Following the stress phase, a recovery period was initiated.

The recovery phase protocol was designed to monitor plant water status and environmental conditions, ensuring uniform recovery conditions for all treatment groups. To mitigate potential positional effects on plant growth, pots were systematically repositioned within the greenhouse on a weekly basis. A standardized sampling protocol was employed for both treatment and control groups to maintain consistency in plant material collection. The recovery phase lasted 15 days for all treatment groups, during which plants were weighed and irrigated to pot capacity every two days. The recovery phase concluded on June 13 for ‘Divadona’ saplings grafted onto Rootpac 20 and on June 21 for those grafted onto Rootpac 40. Leaf samples of 80 plants per treatment were harvested and immediately frozen in liquid nitrogen following both stress and recovery treatments. These samples were stored at −80°C until biochemical analyses were performed. Leaf samples were consistently collected from the third fully expanded leaf from the apex, between 12:00 and 14:00 h, to minimize diurnal variations in biochemical parameters. After the post‐stress sampling, the rootstocks were maintained under optimal growth conditions to assess their recovery capacity over the following 15 days (Figure 1). A comprehensive measurement schedule was established to capture both physiological and morphological parameters at key experimental time points. Physiological measurements were taken immediately following the stress and recovery treatments. Following the recovery period, both physiological and morphological parameters were assessed. Morphological measurements, including plant height, stem diameter, and leaf area, were recorded using standardized techniques, such as digital calipers for stem diameter, a ruler or measuring tape for plant height, and an image analysis software or leaf area meter for leaf area, to ensure data consistency across all treatment groups and replicates.

### 2.2 Morphological measurements

#### 2.2.1 Shoot length and shoot diameter and leaf area

Shoot length and shoot diameter were measured at the beginning of the experiment upon the stress initiation and at the end of the treatment, and therefore the recovery phase, using a ruler and digital caliper, respectively. To eliminate heterogeneity among rootstocks with different growth vigor and to facilitate proportional calculations of the values, the percentage increase was calculated using the formula below (Bolat et al., 2022):

Increase (%) = ((Final value ‐ Initial value) / Initial value) × 100.

At the end of the recovery phase, fully developed leaves from the middle part of the shoot were harvested for leaf area measurements and photographed. Subsequently, the leaf area of all plants in each treatment combination was determined in cm^2^ using the Image J software program. The average number of leaves per plant was calculated by counting the leaves of the plants that were harvested at the end of the experiment (Dogan et al., 2022; Bolat et al., 2022).

#### 2.2.2 Fresh and dry weights of plants and root length

At the end of the experiment, all plants from each treatment were carefully uprooted. The roots were thoroughly washed with tap water to remove any adhering soil. Fresh root weight and fresh shoot weight were determined using an analytical balance. The plants were then subjected to a drying process in an oven at 70°C for 72 h. After drying, the dry weight of the root and shoot samples was measured again using an analytical scales. At the end of the recovery phase, the root length was determined using a ruler (Bolat et al., 2022).

### 2.3 Physiological measurements

To assess the plants' response to stress and their recovery capacity following the treatment phase, several physiological measurements were conducted. All physiological parameters were measured twice, once after the stress phase and again following the recovery phase. The data were then evaluated separately for both phases.

#### 2.3.1 Stomatal conductance

For all plants across different treatments, newly developed leaves from the middle‐upper portion of the shoot were selected. Stomatal conductance measurements were made during daylight hours (12:00–14:00) using a ‘Leaf Porometer' (Decagon Devices Inc., Model SC‐1, Steady‐State Diffusion Porometer) (Dogan et al., 2022).

#### 2.3.2 Chlorophyll content

For all plants from each treatment group, leaves from the middle‐upper part of the shoot were identified, and chlorophyll content was determined using a SPAD‐502 Plus meter (Konica Minolta Optics, Inc.;) (Bolat et al., 2022).

#### 2.3.3 Leaf relative water content (RWC)

To determine the leaf relative water content, fresh leaf samples were collected from each pot and weighed (Fresh Weight, FW). These samples were placed in petri dishes containing 100 mL of distilled water and left for 24 h to determine their turgor weight (TW). Subsequently, the samples were dried in an oven at 65–70°C, and their dry weight (DW) was determined. The relative water content of the leaves was calculated using the formula (Bolat et al., 2022).

$$\mathrm{RWC}=\left(\frac{\mathrm{FW}‐\mathrm{DW}}{\mathrm{TW}‐\mathrm{DW}}\right)\times 100$$

#### 2.3.4 Membrane permeability

At the end of the experiment, 0.5 g of leaf samples from each plant were placed in distilled water and boiled at 40°C to determine the EC₁ value. The same samples were then boiled at 100°C, and the EC₂ values were recorded. The membrane permeability was calculated using the formula (Lutts et al., 1996).

$$\text{\%}\;\text{Membrane permeability}=\left(\frac{{\mathrm{EC}}_{1}}{{\mathrm{EC}}_{2}}\right)\times 100$$

#### 2.3.5 Normalized difference vegetation index (NDVI)

The NDVI values range from 0.00 to 1.00 and were measured using the Trimble GreenSeeker Handheld Crop Sensor (Trimble Navigation Ltd.). Measurements were taken by positioning the sensor horizontally over the pot and ensuring it measured the top of the rootstock for at least 5 seconds. The sensor was held 60 cm above the plant top, and readings were recorded (Bellvert et al., 2018).

#### 2.3.6 Leaf temperature

Leaf temperature was determined using the FLIR T540 thermal camera (FLIR Systems Inc.,). The camera, mounted on a tripod at a fixed distance and calibrated for emission, captured images of the plants. Using the FLIR Tools software, average temperature readings from the images were calculated. To obtain a homogeneous temperature reading, measurements were taken from the lower, middle, and upper sections of the plant, and the averages were computed (Kaya et al., 2024).

### 2.4 Biochemical analyses

#### 2.4.1 Determination of proline, malondialdehyde (MDA), H_2_O_2_ content, and antioxidant enzymes in leaves

The proline concentration in the treatment samples was measured following a slightly modified version of Kaya (2020)‘s method. To do so, 0.5 g of leaf tissue was homogenized in 10 mL of 3% sulfosalicylic acid. The mixture was then centrifuged at 10,000 rpm for 10 minutes. A reaction solution was prepared by mixing 2 mL of the supernatant with 2 mL of glacial acetic acid and 2 mL of freshly prepared acid‐ninhydrin. The reaction occurred by incubating the tubes in a 90°C water bath for 1. Afterwards, 5 mL of toluene was added to extract the product. The absorbance of the toluene phase was measured at 520 nm using a spectrophotometer (Shimadzu UV‐1700). Proline content was calculated by comparing the absorbance readings with a standard curve generated using L‐proline, and the results were expressed as μg g^−1^ fresh weight.

The malondialdehyde (MDA) content was determined with slight modifications to the method outlined by Rende et al. (2018). In our approach, 0.5 g of leaf tissue was homogenized in trichloroacetic acid (TCA). Following centrifugation at 10 000 g for 5 minutes, the reaction mixture consisting of thiobarbituric acid (TBA) and TCA was added to the supernatant and heated at 95°C for 45 minutes. After cooling, the mixture was centrifuged at 10 000 g for 15 minutes. Absorbance values of the supernatant were measured at 532 nm and 600 nm. The MDA content was expressed in nmol g^−1^ fresh weight.

Hydrogen peroxide (H_2_O_2_) content was determined using the eFOX reagent, based on the methodology of Kaya and Kose (2017). Leaf samples were extracted with cold acetone containing 25 mM H₂SO₄ and centrifuged at 3000 g for 5 minutes at 4°C. The supernatant (50 μL) was mixed with 950 μL of the eFOX reagent (containing 250 μM ferroammonium sulfate, 1% ethanol, 100 μM sorbitol, and 100 μM xylenol orange) and incubated at room temperature for 30 minutes. Absorbance was measured at 550 and 800 nm, and the H₂O₂ concentration was calculated from a standard curve prepared with known H₂O₂ amounts.

The activities of antioxidant enzymes, including superoxide dismutase (SOD), peroxidase (POD), and catalase (CAT), were assessed with minor adjustments to the methods described by Rende et al. (2018) and Kaya and Kose (2017). Leaf samples (0.5 g) were homogenized in 3 mL of 50 mM phosphate buffer (pH 7). After centrifugation at 15 000 × g for 15 minutes at 4°C, the supernatant was stored at −80°C. For enzyme assays, the supernatant was extracted using a 0.1 mM phosphate buffer (pH 7.8) containing 0.5% polyvinylpyrrolidone (PVP), 1 mM phenylmethylsulfonyl fluoride (PMSF), and 1 mM ethylenediaminetetraacetic acid (EDTA). The activities of SOD, POD, and CAT were determined by measuring absorbance at 560, 470, and 240 nm, respectively. One unit (U) of SOD activity was defined as the amount of enzyme that caused a 50% reduction in absorbance. One unit of POD and CAT activity was determined as the amount of enzyme that increased absorbance by 0.01 per minute.

#### 2.4.2 Hormone analysis of leaves

The extraction and purification of hormones, including cytokinins, indole‐3‐acetic acid (IAA), gibberellic acid (GA), and abscisic acid (ABA), were conducted with slight modifications to the method of Koshita et al. (1999). A 1 g fresh leaf sample was first homogenized with 80% methanol at −40°C for 10 minutes, after which the supernatant was filtered. The resulting mixture was dried at 35°C and re‐dissolved in a 0.1 M KH₂PO₄ solution (pH 8.0). Separation of hormones was carried out using a Sep‐Pak C‐18 (Waters) cartridge. Hormone concentrations were determined by measuring absorbance at 265 nm using high‐performance liquid chromatography (HPLC) with a UV detector (Agilent Technologies).

#### 2.4.3 Sugar accumulation in leaves

To extract glucose, fructose, and sucrose, 1 g of pulverized fresh leaf tissue was incubated in 10 mL water at 70°C for 12 hours. The supernatant was stored at −20°C for later analysis. To determine the dry weight of the leaves, another 1 g sample was dried at 60°C for 96 hours. The extracts were filtered through a 0.2 μm membrane and appropriately diluted. Sugar quantification was performed using a Waters HPLC system, consisting of a Waters 600S controller, a Waters 626 pump, and a Waters 717 autosampler. A 10–90 μL sample volume was injected, and the separation was carried out using a CarboPac PA‐100 analytical column (Dionex Pty Ltd) with a guard column. The mobile phase was a gradient of sodium hydroxide (50–150 mM). The sugars were detected using pulsed amperometric detection with a Waters 464 detector. Quantification was based on standard curves generated from external standards analyzed before and after every 10 samples to ensure accuracy and account for potential instrument drift (Albertson and Grof, 2007).

#### 2.4.4 Mineral analysis of leaves

After the leaves were harvested, they were washed with distilled water, dried on blotting paper, and placed in an oven at 65–70°C for 48 hours. Once dried, the samples were ground in a mortar. The nitrogen (N) content was determined using the Kjeldahl method (Kacar and Inal, 2008; Ates and Kaya, 2021), while magnesium (Mg), calcium (Ca), and potassium (K) concentrations were analyzed using an atomic absorption spectrophotometer. Phosphorus (P) content was measured using spectrophotometry (Shimadzu UV‐1700). The macro‐ and microelements were expressed as percentages (Kacar and Inal, 2008; Ates and Kaya, 2021).

### 2.5 Data analysis

In our study, data obtained from the measurements of morphological, physiological, and biochemical parameters were analyzed using the Origin Pro statistical software (Origin 2024 version). The mean values of the variables for each treatment group were compared using Tukey's HSD variance analysis (Two‐Way ANOVA). As indicated by ANOVA, significant differences between the cultivar‐treatment groups were further evaluated by Tukey's HSD test (*p* ≤ 0.01) to separate the means. Network correlation analysis was performed using the Python programming language with the Pandas, NetworkX, and Matplotlib libraries. Additionally, hierarchical clustering analysis (HCA) was conducted through the online data visualization platform, SRplot (http://www.bioinformatics.com.cn/srplot).

## 3 RESULTS

### 3.1 Effects of drought and heat stress on selected physiological parameters of rootstocks grafted with the ‘Divadona’ peach cultivar

Our statistical analysis revealed significant differences (p ≤ 0.01) for cultivar, treatment, and their interactions across all measured parameters (Table 1). Under control conditions, Rootpac 20/‘Divadona’ exhibited a relative shoot diameter (RSD) of 21.30%, while Rootpac 40/‘Divadona’ demonstrated a higher RSD of 24.08%. Drought stress resulted in a reduction of RSD in both rootstocks, with Rootpac 20/‘Divadona’ and Rootpac 40/‘Divadona’ decreasing to 16.44% and 21.07%, respectively. Similarly, heat stress led to reductions in RSD, recorded at 19.47% for Rootpac 20/‘Divadona’ and 22.23% for Rootpac 40/‘Divadona’. The combined drought and heat stress treatment induced further declines, reaching 14.99% in Rootpac 20/‘Divadona’ and 19.75% in Rootpac 40/‘Divadona’. Relative shoot length (RSL) followed a similar trend, with Rootpac 20/‘Divadona’ and Rootpac 40/‘Divadona’ exhibiting 34.94% and 44.60% under control conditions, respectively. Drought stress resulted in a decline of RSL to 27.11% in Rootpac 20/‘Divadona’ and 39.81% in Rootpac 40/‘Divadona’, while heat stress led to reductions of 30.46% and 41.16%, respectively. The combined stress application had the most pronounced effect, reducing RSL to 24.77% in Rootpac 20/‘Divadona’ and 37.22% in Rootpac 40/‘Divadona’. Leaf area (LA) was measured at 102.75 cm^2^ for Rootpac 20/‘Divadona’ and 121.09 cm^2^ for Rootpac 40/‘Divadona’ under control conditions. Drought stress led to reductions of 8.5% and 8.7% in Rootpac 20/‘Divadona’ and Rootpac 40/‘Divadona’, respectively. Heat stress caused similar declines, with decreases of 8.9% in Rootpac 20/‘Divadona’ and 3.0% in Rootpac 40/‘Divadona’. The combined stress treatment resulted in the most substantial reductions, with LA declining by 15.4% in Rootpac 20/‘Divadona’ and 11.6% in Rootpac 40/‘Divadona’ compared to their respective controls. Leaf number (LN) was highest under control conditions, reaching 73.15 in Rootpac 20/‘Divadona’ and 69.97 in Rootpac 40/‘Divadona’. Drought stress led to reductions of 14.7% and 14.0% in Rootpac 20/‘Divadona’ and Rootpac 40/‘Divadona’, respectively. Under heat stress, LN decreased by 5.4% in Rootpac 20/‘Divadona’ and 6.1% in Rootpac 40/‘Divadona’. The combined stress treatment resulted in the most significant decreases, with reductions of 20.7% and 18.5% in Rootpac 20/‘Divadona’ and Rootpac 40/‘Divadona’, respectively.

**TABLE 1:** Effects of drought and heat stress on physiological properties of rootstocks grafted onto the‘Divadona’ peach cultivar.

Cultivar	Application	RSD (%)	RSL (%)	LA (cm^2^)	LN	RL (cm)	FSW (g plant^−1^)	FRW (g plant^−1^)	DSW (g plant^−1^)	DRW (g plant^−1^)
Rootpac 20/ Divadona	Control	21.30b	34.94c	102.75c	73.15a	37.89b	101.33a	56.89a	44.44a	23.55a
	Drought	16.44c	27.11de	94.00d	62.34c	35.80bc	91.31b	50.24b	41.07ab	20.70bc
	Heat Shock	19.47bc	30.46d	93.67d	69.19b	39.41ab	92.81b	54.61ab	38.20b	21.46b
	Drought + Heat Shock	14.99 cd	24.77ef	86.99e	58.01d	33.20c	81.87 cd	46.47c	35.40bc	19.27c
Rootpac 40/ Divadona	Control	24.08a	44.60a	121.09a	69.97b	46.40a	93.70b	55.64a	38.06b	23.10a
	Drought	21.07b	39.81b	110.55b	60.16 cd	39.20ab	88.03bc	53.54ab	34.49c	21.98b
	Heat Shock	22.23ab	41.16ab	117.50ab	65.71bc	45.65a	91.34b	54.55ab	36.79bc	22.04ab
	Drought + Heat Shock	19.75bc	37.22bc	107.06bc	56.99de	44.47ab	84.24c	50.43b	33.81c	21.44b
Cultivar		**	**	**	**	**	**	**	**	**
Application		**	**	**	**	**	**	**	**	**
Cultivar *x* Application		**	**	**	**	**	**	**	**	**

Notes: Dry shoot weight (DSW), Dry root weight (DRW), Fresh root weight (FRW), Fresh shoot weight (FSW), Leaf area (LA), Leaf number (LN), Root length (RL), Relative shoot diameter (RSD), and Relative shoot length (RSL), **Significance level: Different letters within the same column signify significant differences between treatments at p ≤ 0.01 (Tukey's HSD test).

In our results, root length (RL) was measured at 37.89 cm for Rootpac 20/‘Divadona’ and 46.40 cm for Rootpac 40/‘Divadona’ under control conditions. Drought stress led to reductions of 5.5% and 15.5% in Rootpac 20/‘Divadona’ and Rootpac 40/‘Divadona’, respectively. Heat stress had varying effects, with RL increasing by 4.0% in Rootpac 20/‘Divadona’ but decreasing by 1.6% in Rootpac 40/‘Divadona’. The combined stress treatment resulted in declines of 12.3% and 4.1% in Rootpac 20/‘Divadona’ and Rootpac 40/‘Divadona’, respectively. Fresh shoot weight (FSW) under control conditions was 101.33 g plant^−1^ for Rootpac 20/‘Divadona’ and 93.70 g plant^−1^ for Rootpac 40/‘Divadona’. Drought stress resulted in reductions of 9.9% and 6.1%, while heat stress caused declines of 8.4% and 2.5% in Rootpac 20/‘Divadona’ and Rootpac 40/‘Divadona’, respectively. The combined stress treatment led to reductions of 19.2% in Rootpac 20/‘Divadona’ and 10.1% in Rootpac 40/‘Divadona’. Fresh root weight (FRW) exhibited similar responses, with control values of 56.89 g plant^−1^ in Rootpac 20/‘Divadona’ and 55.64 g plant^−1^ in Rootpac 40/‘Divadona’. Drought stress led to reductions of 11.7% and 3.8%, while heat stress caused declines of 4.0% and 2.0% in Rootpac 20/‘Divadona’ and Rootpac 40/‘Divadona’, respectively. The combined stress treatment resulted in the largest declines, with FRW decreasing by 18.3% in Rootpac 20/‘Divadona’ and 9.4% in Rootpac 40/‘Divadona’. Dry shoot weight (DSW) and dry root weight (DRW) followed similar trends. Under control conditions, DSW was recorded at 44.44 g plant^−1^ for Rootpac 20/‘Divadona’ and 38.06 g plant^−1^ for Rootpac 40/‘Divadona’. Drought stress led to reductions of 7.6% and 9.4%, while heat stress resulted in declines of 14.1% and 3.3%, respectively. The combined stress treatment had the most pronounced effects, leading to reductions of 20.3% in Rootpac 20/‘Divadona’ and 11.2% in Rootpac 40/‘Divadona’. DRW under control conditions was 23.55 g plant^−1^ for Rootpac 20/‘Divadona’ and 23.10 g plant^−1^ for Rootpac 40/‘Divadona’. Drought stress led to reductions of 12.1% and 4.8%, while heat stress resulted in declines of 8.9% and 4.6%, respectively. The combined stress treatment led to decreases of 18.2% in Rootpac 20/‘Divadona’ and 7.2% in Rootpac 40/‘Divadona’.

### 3.2 Physiological responses of the ‘Divadona’ peach cultivar to stress and recovery

**FIGURE 2:**
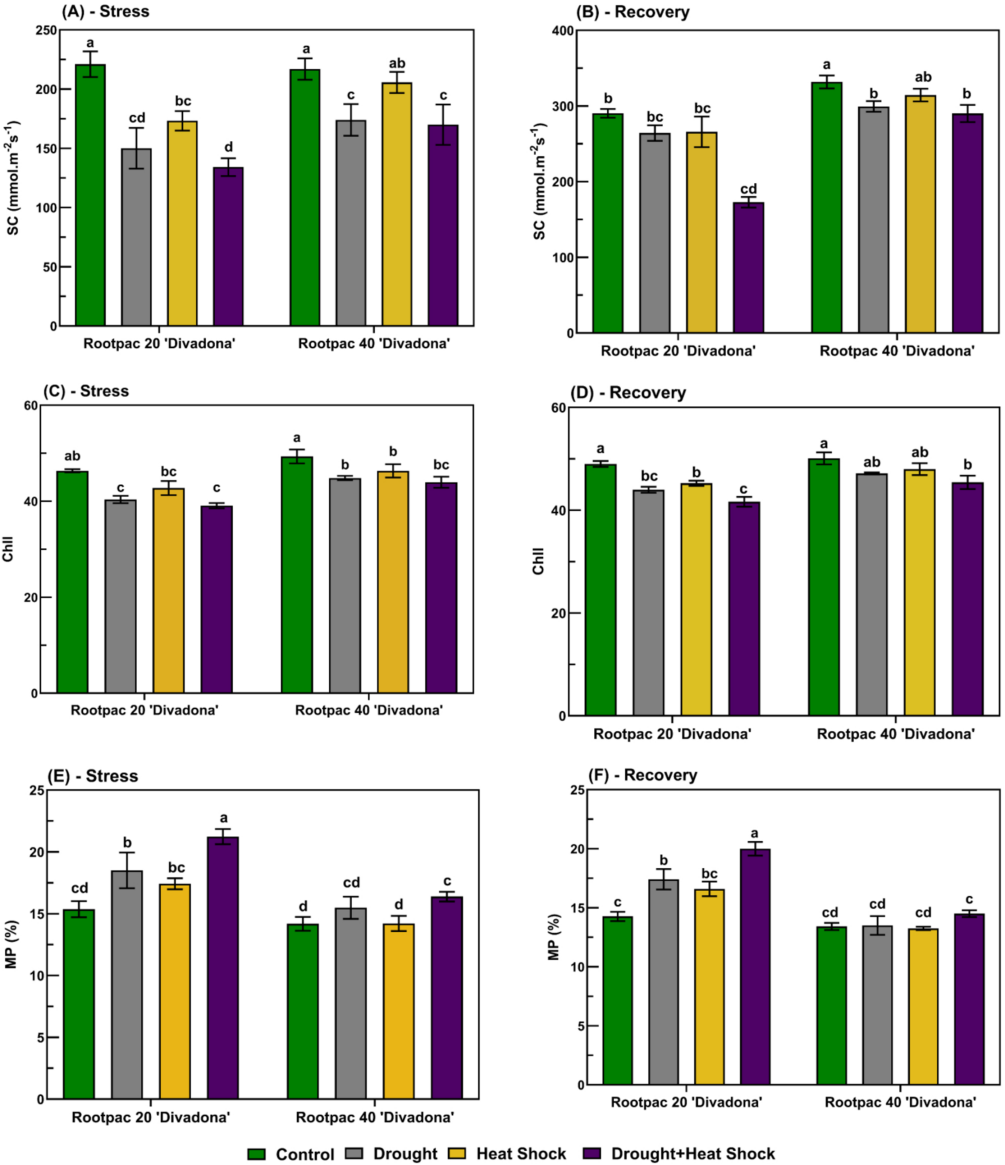
Physiological responses of the ‘Divadona’ peach cultivar to stress and recovery conditions. (A‐B) Stomatal Conductance (SC), (C‐D) Chlorophyll Content (Chll), (E‐F) Membrane Permeability (MP). Bars assigned different low‐case letters indicate a statistically significant difference. Tukey's multiple range test at significance level of *p* ≤ 0.01.

**FIGURE 3:**
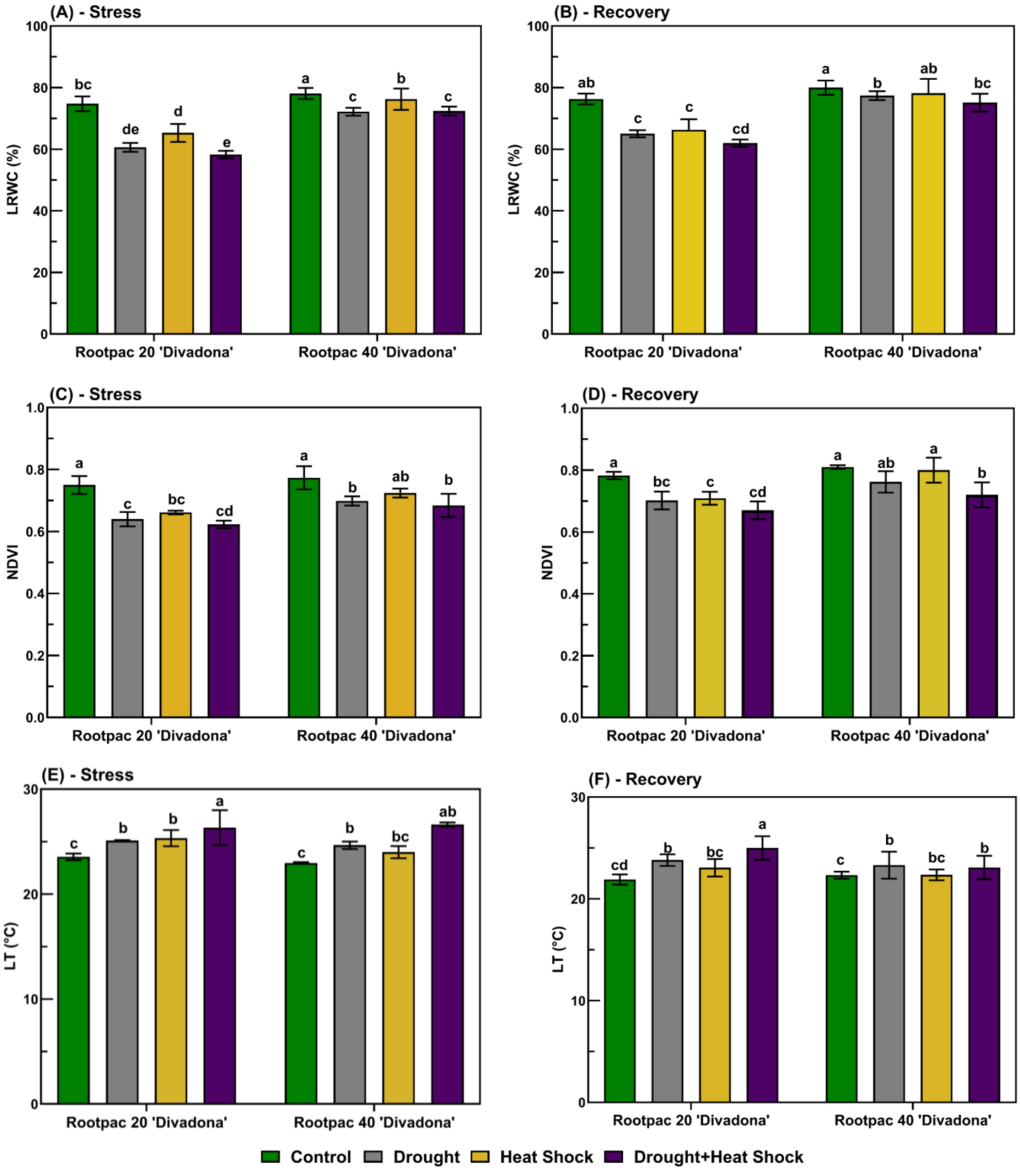
Physiological responses of the ‘Divadona’ peach cultivar to stress and recovery conditions. (A‐B) Leaf Relative Water Content (LRWC), (C‐D) Normalized Difference Vegetation Index (NDVI), (E‐F) Leaf Temperature (LT). Bars assigned different low‐case letters indicate a statistically significant difference. Tukey's multiple range test at significance level of *p* ≤ 0.01.

Our results confirmed that all physiological parameters exhibited significant differences (*p* ≤ 0.01) among treatments and across time points (Figure 2A‐E and Figure 3A‐E). Under control conditions, stomatal conductance (SC), was measured at 210.4 mmol m^−2^ s^−1^. Drought stress led to a significant reduction, with SC decreasing to 95.2 mmol m^−2^ s^−1^ (a 54.7% decrease) by the end of the stress period. Heat stress resulted in a slightly less pronounced decrease, reaching 120.8 mmol m^−2^ s^−1^ (42.6% decrease). The combined drought and heat stress treatment caused the most severe decline, with SC decreasing to 78.5 mmol m^−2^ s^−1^, representing a 62.7% decrease compared to the control. During the recovery period, SC partially improved, reaching 165.3 mmol m^−2^ s^−1^ in drought‐stressed plants, 182.1 mmol m^−2^ s^−1^ in heat‐stressed plants, and 140.7 mmol m^−2^ s^−1^ in plants subjected to combined stress. However, none of the treatments fully recovered to control levels. On the other hand, chlorophyll content (Chll) under control conditions was recorded at 42.7 SPAD units. Drought stress reduced chlorophyll levels to 34.1 SPAD units (20.1% decrease), while heat stress resulted in a decrease to 36.8 SPAD units (a 13.8% reduction). The most substantial decline was observed under combined drought and heat stress, where chlorophyll content decreased to 30.9 SPAD units, corresponding to a 27.6% decrease compared to the control. Recovery led to a partial increase in chlorophyll content, with values reaching 39.4 SPAD units in drought‐stressed plants, 40.5 SPAD units in heat‐stressed plants, and 35.8 SPAD units in plants exposed to combined stress. In our results, membrane permeability (MP) in control plants was measured at 24.5%. Drought stress caused MP to rise to 41.8%, marking a 70.6% increase. Heat stress also led to a significant increase, with MP reaching 38.6% (57.6% increase). The combined stress treatment resulted in the highest MP values, reaching 47.2%, reflecting a 92.6% increase relative to the control. During the recovery period, MP levels decreased but did not return to baseline, with final values of 32.5% for drought‐stressed plants, 30.8% for heat‐stressed plants, and 38.1% for those subjected to combined stress.

Under control conditions, leaf relative water content (LRWC) was recorded at 85.7%. Drought stress significantly reduced LRWC to 61.2%, representing a 28.6% decrease. Heat stress led to a decrease to 72.5% (a 15.4% decrease), while the combined drought and heat stress treatment resulted in the lowest LRWC values, decreasing to 55.8%, reflecting a 34.9% decrease compared to the control. During the recovery phase, LRWC partially increased, reaching 78.1% in drought‐stressed plants, 81.4% in heat‐stressed plants, and 70.3% in plants exposed to combined stress. However, full recovery was not observed in any treatment. On the other side, normalized difference vegetation index (NDVI) values under control conditions were measured at 0.83. Drought stress caused a significant decline to 0.65 (21.7% decrease), while heat stress led to a reduction to 0.71 (14.5% decrease). The combined drought and heat stress treatment resulted in the lowest NDVI values, decreasing to 0.60, corresponding to a 27.7% decrease relative to the control. After the recovery period, NDVI improved, with values increasing to 0.77 for drought‐stressed plants, 0.79 for heat‐stressed plants, and 0.68 for those subjected to combined stress. In our findings, leaf temperature (LT) was also recorded at 28.4°C under control conditions. Drought stress led to a significant increase, reaching 34.7°C (22.2% increase). Heat stress caused an even greater increase in LT, with values rising to 38.1°C (34.2% increase). The combined drought and heat stress treatment resulted in the highest LT values, reaching 40.3°C, reflecting a 41.9% increase compared to the control. During recovery, LT decreased but did not return to baseline levels, with final values of 30.9°C in drought‐stressed plants, 31.8°C in heat‐stressed plants, and 35.2°C in plants exposed to combined stress.

**FIGURE 4:**
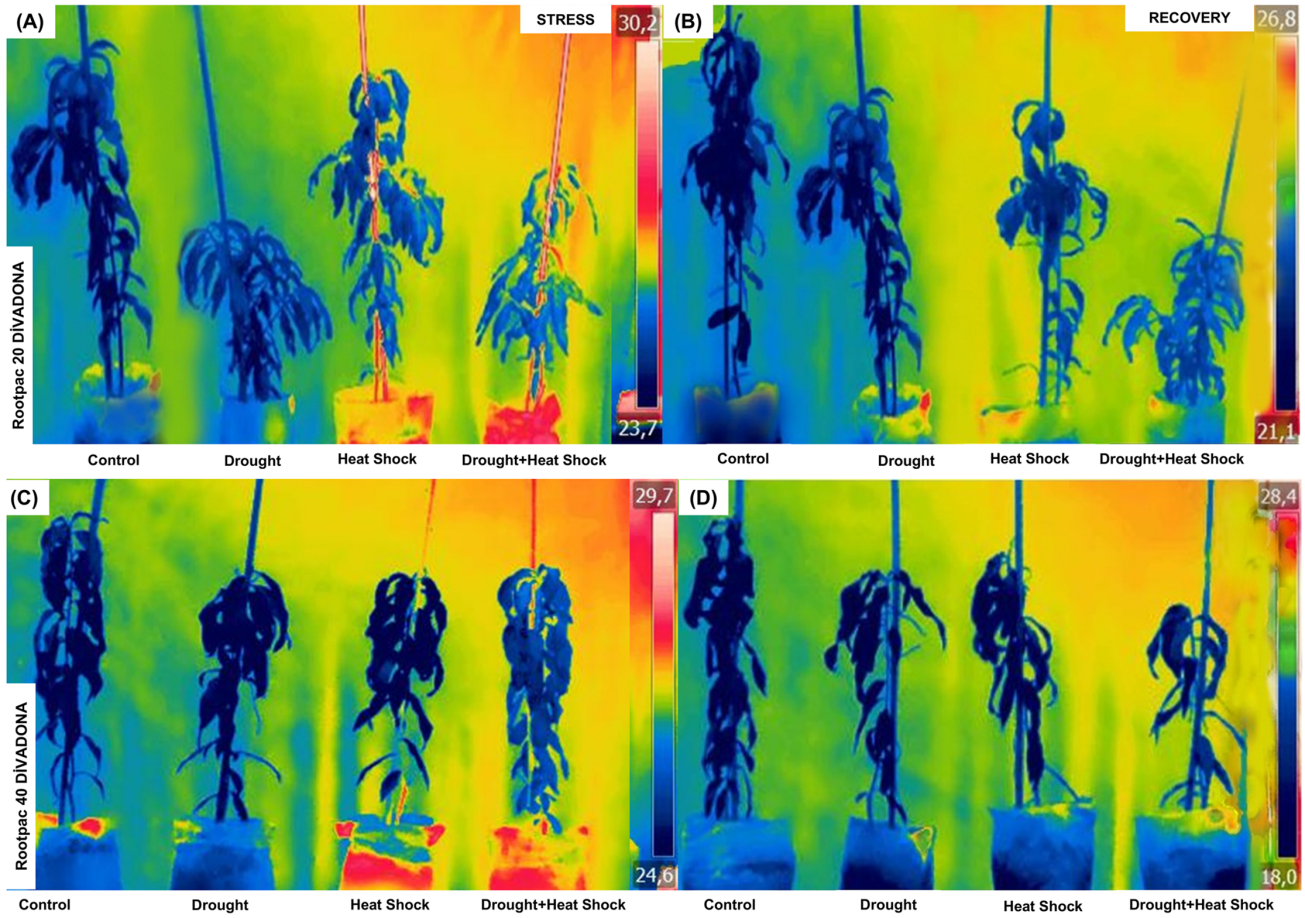
Thermal Imaging Analysis of the ‘Divadona’ Peach Cultivar Under Different Stress and Recovery Conditions (Rootpac20/‘Divadona’, A‐B; Rootpac40/‘Divadona’ C‐D).

The thermal images in Figure 4 indicated temperature distributions in the ‘Divadona’ peach cultivar grafted onto Rootpac 20/‘Divadona’ and Rootpac 40/‘Divadona’ rootstocks under drought stress, heat shock, their combination, and during recovery periods. The images utilized a color scale from blue (lower temperatures) to red (higher temperatures) to illustrate temperature variations. Under control conditions, both rootstocks exhibited predominantly dark blue colorations, indicating lower leaf temperatures. Drought stress induced a transition towards warmer colors, with plants displaying light blue and yellow hues. Heat shock further intensified these thermal changes, as evidenced by the presence of yellow‐green and red areas in the plants. The combination of drought and heat shock resulted in the most pronounced thermal responses, with red, yellow, and light blue hues indicating the highest temperatures observed among all treatments. Thermal variations between the two rootstocks were evident. During the stress period, Rootpac 40/‘Divadona’ retained more blue coloration compared to Rootpac 20/‘Divadona’, suggesting relatively lower leaf temperatures under stress conditions. In contrast, Rootpac 20/‘Divadona’ exhibited a more pronounced shift towards warmer colors, particularly in the drought + heat shock treatment. During the recovery period, thermal images of both rootstocks showed a trend toward cooler colors compared to the stress phase. However, previously stressed plants still exhibited warmer hues relative to control plants.

### 3.3 Biochemical responses of the ‘Divadona’ peach cultivar to stress and recovery

#### 3.3.1 Oxidative stress and antioxidant enzyme activity

**FIGURE 5:**
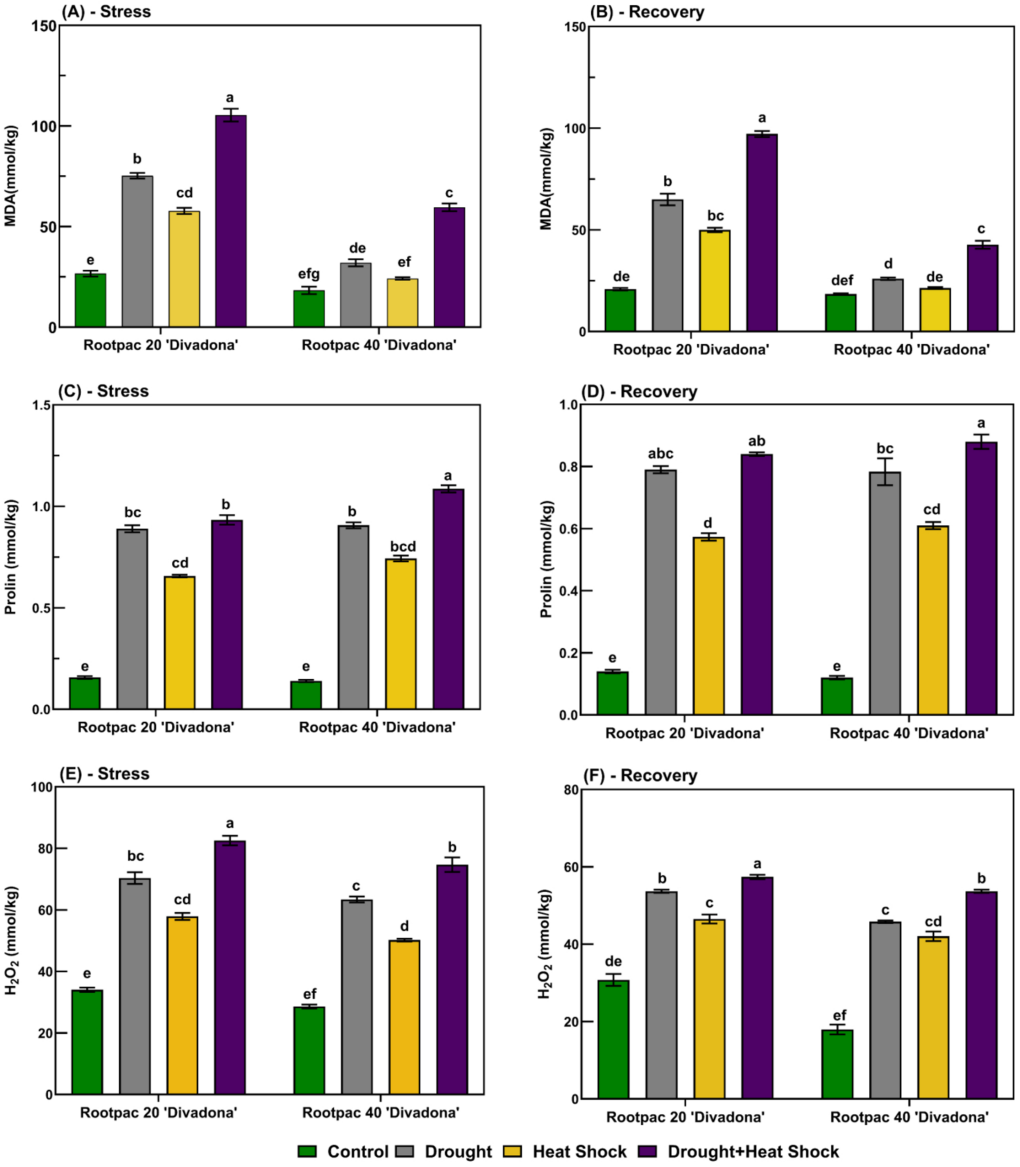
Physiological responses of ‘Divadona’ peach cultivar to drought and heat stress: changes in (A‐B) Malondialdehyde (MDA), (C‐D) Proline levels and Hydrogen Peroxide (H₂O₂), (E‐F) during stress and recovery. Bars assigned different low‐case letters indicate a statistically significant difference. Tukey's multiple range test at significance level of p ≤ 0.01.

**FIGURE 6:**
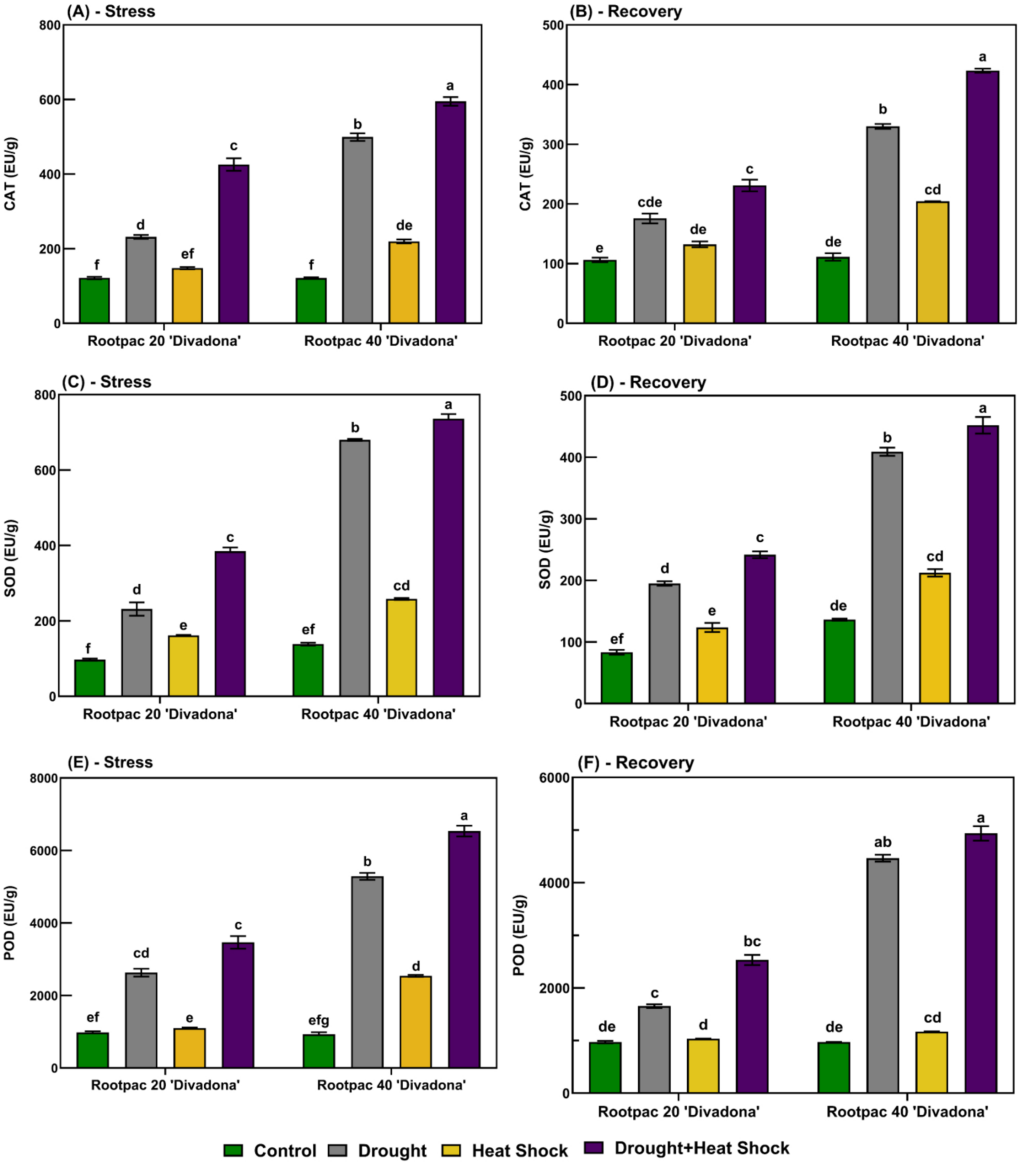
Antioxidant responses of ‘Divadona’ peach cultivar to drought and heat stress: changes in (A‐B) Catalase (CAT), (C‐D) Superoxide Dismutase (SOD) and (E‐F) Peroxidase (POD) levels during stress and recovery. Bars assigned different low‐case letters indicate a statistically significant difference. Tukey's multiple range test at significance level of p ≤ 0.01.

During the stress period, malondialdehyde (MDA) content (Figure 5A‐B) increased significantly, with Rootpac 20/‘Divadona’ exhibiting the highest accumulation under combined drought and heat shock (~110 nmol g^−1^), nearly five times higher than the control (~22 mmol/kg^−1^). Rootpac 40/‘Divadona’ showed lower MDA accumulation (~55 mmol/kg under combined stress). During recovery, MDA content decreased but remained elevated, particularly in Rootpac 20/‘Divadona’ (~100 mmol/kg under combined stress). Proline content (Figure 5C‐D) increased under stress, reaching ~1.1 mmol/kg in Rootpac 20/‘Divadona’ and ~ 0.9 mmol/kg in Rootpac 40/‘Divadona’ under combined stress, representing a four‐ to fivefold rise compared to controls (~0.2 mmol/kg). During recovery, proline levels declined but remained higher than controls. H₂O₂ levels (Figure 5E‐F) peaked under combined stress (~95 mmol kg^−1^ in Rootpac 20/‘Divadona’, ~85 mmol kg^−1^ in Rootpac 40/‘Divadona’), nearly threefold higher than controls (~30 mmol kg^−1^). Recovery led to partial decreases, but stressed plants still exhibited elevated H₂O₂ (~60 mmol kg^−1^ in Rootpac 20/‘Divadona’, ~55 mmol kg^−1^ in Rootpac 40/‘Divadona’). During stress, on the other hand, catalase (CAT) activity increased significantly under combined drought and heat stress (Figure 6A‐B). In Rootpac 20/‘Divadona’, control plants exhibited 90 EU g^−1^, while combined stress increased activity to 600 EU g^−1^ (566.7% rise). Rootpac 40/‘Divadona’ showed a similar trend, with CAT activity reaching 620 EU g^−1^ (520% increase from control). During recovery, CAT activity declined but remained elevated. In Rootpac 20/‘Divadona’, the highest activity was recorded under heat stress (280 EU g^−1^, 211.1% increase), while combined stress resulted in 320 EU g^−1^ (255.6% increase). In Rootpac 40/‘Divadona’, combined stress maintained the highest activity (420 EU g^−1^, 320% increase; Figure 6B). Under stress, superoxide dismutase (SOD) activity increased markedly (Figure 6C‐D). In Rootpac 20/‘Divadona’, control plants had 110 EU g^−1^, while combined stress led to 800 EU g^−1^ (627.3% increase). Rootpac 40/‘Divadona’ exhibited a similar pattern, with a peak of 850 EU g^−1^. During recovery, activity decreased but remained higher than the control. In Rootpac 20/‘Divadona’, combined stress resulted in 450 EU g^−1^ (350% increase). In Rootpac 40/‘Divadona’, the highest activity was recorded under combined stress (470 EU g^−1^, 327.3% increase; Figure 6C‐D). Peroxidase (POD) activity showed the greatest increases under stress. In Rootpac 20/‘Divadona’, control plants exhibited 900 EU g^−1^, while combined stress led to 6500 EU g^−1^. Rootpac 40/‘Divadona’ peaked at 7000 EU g^−1^ (Figure 6C). During recovery, POD activity remained significantly elevated. In Rootpac 20/‘Divadona’, combined stress resulted in 4800 EU g^−1^, while in Rootpac 40/‘Divadona’, the highest activity under combined stress reached 5100 EU g^−1^ (Figure 6E‐F).

#### 3.3.2 Sugar accumulation

**FIGURE 7:**
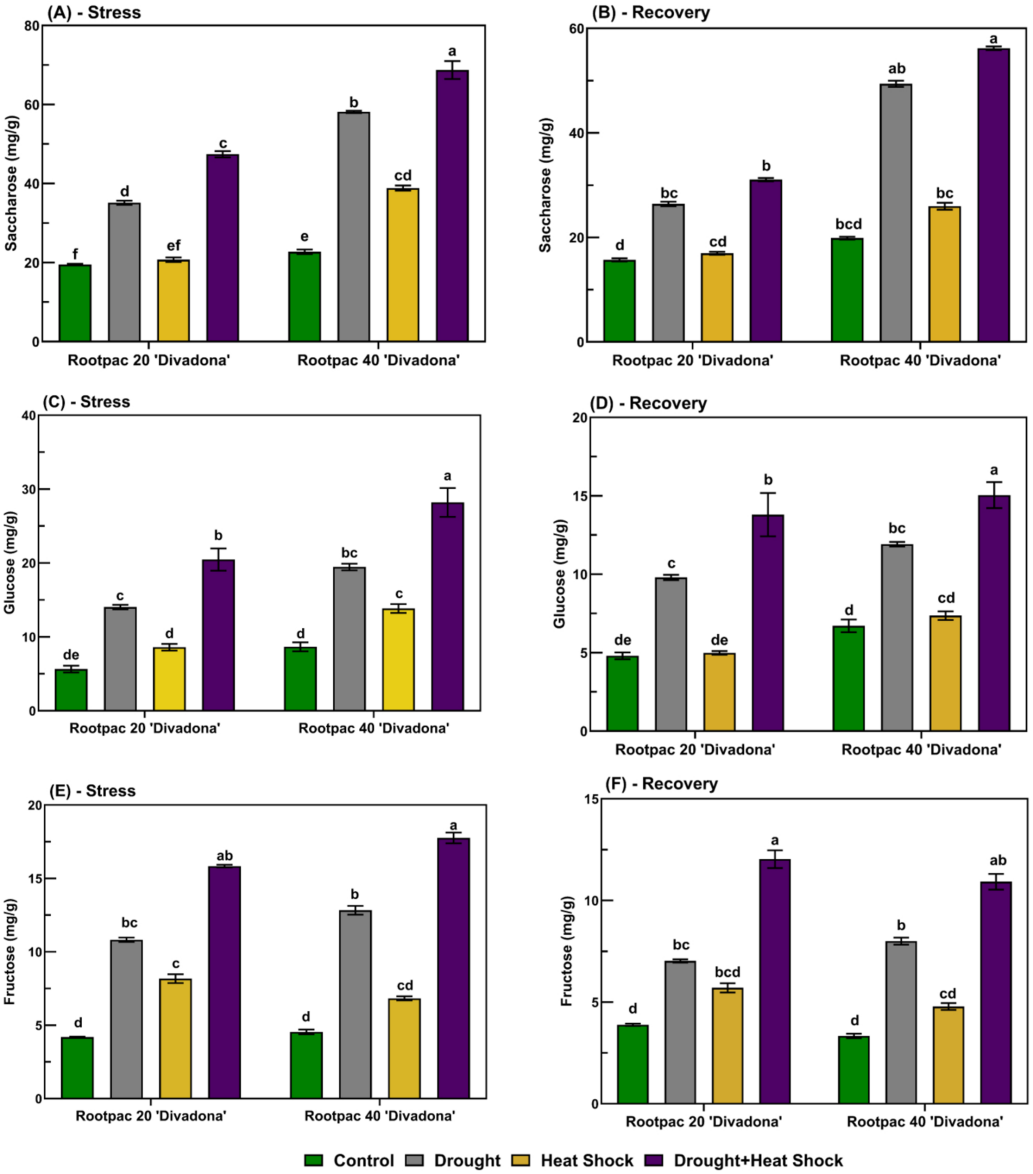
Alterations in soluble sugar composition (sucrose, glucose, and fructose) in the ‘Divadona’ peach cultivar under stress conditions and recovery phases. (A‐B) Sucrose (Saccharose), (C‐D) Glucose, (E‐F) Fructose. Bars assigned different low‐case letters indicate a statistically significant difference. Tukey's multiple range test at significance level of p ≤ 0.01.

Under stress, saccharose content increased significantly, particularly under combined drought and heat stress (Figure 7A‐B). In Rootpac 20/‘Divadona’, control plants had approximately 15 mg g^−1^ saccharose, while combined stress resulted in 60 mg g^−1^ (300% increase). In Rootpac 40/‘Divadona’, saccharose peaked at 70 mg g^−1^ under combined stress, a 366.7% rise from the control (15 mg g^−1^). During recovery, saccharose levels declined but remained higher than the control. In Rootpac 20/‘Divadona’, the highest value was recorded under drought stress (35 mg g^−1^, 133.3% increase), while in Rootpac 40/‘Divadona’, combined stress maintained the highest saccharose content (60 mg g^−1^, 300% increase). Glucose content increased under stress, with the highest accumulation under combined stress (Figure 7C‐D). In Rootpac 20/‘Divadona’, control plants exhibited 5 mg g^−1^ glucose, while combined stress led to 30 mg g^−1^ (500% increase). Rootpac 40/‘Divadona’ followed a similar trend, reaching 32 mg g^−1^ under combined stress (540% increase from the control). During recovery, glucose levels decreased but remained elevated. In Rootpac 20/‘Divadona’, combined stress resulted in 16 mg g^−1^ (220% increase), while in Rootpac 40/‘Divadona’, the highest content was also under combined stress (17 mg g^−1^, 240% increase). Fructose content followed a similar pattern (Figure 7E‐F). Under stress, Rootpac 20/‘Divadona’ control plants had 4 mg g^−1^, while combined stress increased it to 16 mg g^−1^ (300% increase). In Rootpac 40/‘Divadona’, fructose peaked at 18 mg g^−1^ under combined stress, a 350% increase from the control (4 mg g^−1^). During recovery, fructose levels remained higher than the control. In Rootpac 20/‘Divadona’, the highest level was recorded under combined stress (13 mg g^−1^, 225% increase). In Rootpac 40/‘Divadona’, fructose content reached 12 mg g^−1^ under combined stress (200% increase).

#### 3.3.3 Phytohormone dynamics

Our findings indicated that ABA levels significantly increased under stress conditions, whereas GA, IAA, and cytokinin levels exhibited a significant decrease, with partial recovery observed during the recovery phase (Table 2). Under stress conditions, abscisic acid (ABA) levels increased significantly in both rootstocks, particularly under combined drought and heat shock. In Rootpac 20/‘Divadona’, ABA content rose from 170.33 ng g^−1^ D.W in the control to 464.00 ng g^−1^ D.W under combined stress (172.4% increase). Similarly, in Rootpac 40/‘Divadona’, ABA peaked at 939.00 ng g^−1^ D.W under combined stress, a 207.0% increase from the control (306.00 ng g^−1^ D.W). During recovery, ABA levels decreased but remained elevated compared to the control. In Rootpac 20/‘Divadona’, ABA content was 291.00 ng g^−1^ D.W under combined stress (71.0% higher than control), while in Rootpac 40/‘Divadona’, it reached 508.33 ng g^−1^ D.W, reflecting a 121.7% increase over the control (229.33 ng g^−1^ D.W). Gibberellic Acid (GA) content declined under stress, with the lowest levels recorded under combined drought and heat shock. In Rootpac 20/‘Divadona’, GA decreased from 3.69 ng g^−1^ D.W in the control to 1.15 ng g^−1^ D.W under combined stress (68.8% decrease). In Rootpac 40/‘Divadona’, GA content decreased from 4.44 ng g^−1^ D.W (control) to 2.57 ng g^−1^ D.W under combined stress (42.1% decrease). During recovery, GA levels showed partial recovery but remained lower than the control. In Rootpac 20/‘Divadona’, GA content reached 2.06 ng g^−1^ D.W under combined stress (44.2% decrease from the control), while in Rootpac 40/‘Divadona’, GA was 3.33 ng g^−1^ D.W, 47.6% lower than the control (6.36 ng g^−1^ D.W). Indole‐3‐Acetic Acid (IAA) content also decreased under stress, with the lowest values observed under combined drought and heat shock. In Rootpac 20/‘Divadona’, IAA declined from 2.29 ng mg^−1^ tissue (control) to 0.86 ng mg^−1^ tissue under combined stress (62.4% reduction). In Rootpac 40/‘Divadona’, IAA decreased from 3.64 ng mg^−1^ tissue to 1.95 ng mg^−1^ tissue under combined stress (46.4% decrease). During recovery, IAA levels showed partial recovery. In Rootpac 20/‘Divadona’, IAA was 1.22 ng mg^−1^ tissue under combined stress (46.7% lower than the control), while in Rootpac 40/‘Divadona’, IAA reached 2.57 ng mg^−1^ tissue, a 48.4% decrease from the control (4.98 ng mg^−1^ tissue). Cytokinin levels followed a decreasing trend under stress. In Rootpac 20/‘Divadona’, content declined from 2.56 ng g^−1^ D.W (control) to 1.16 ng g^−1^ D.W under combined stress (54.7% decrease). In Rootpac 40/‘Divadona’, cytokinin levels decreased from 3.84 ng g^−1^ D.W (control) to 1.12 ng g^−1^ D.W under combined stress (70.8% decrease). During recovery, cytokinin levels remained lower than the control. In Rootpac 20/‘Divadona’, combined stress resulted in 1.48 ng g^−1^ D.W, a 56.5% reduction from the control (3.40 ng g^−1^ D.W). In Rootpac 40/‘Divadona’, cytokinin content reached 2.86 ng g^−1^ D.W under combined stress, marking a 42.4% decrease compared to the control (4.97 ng g^−1^ D.W).

**TABLE 2:** The hormone content during stress and recovery in the leaves of the ‘Divadona’ peach cultivar.

		Stress				Recovery			
Cultivar	Application	ABA (ng g^−1^DW)	GA (ng g^−1^DW)	IAA (ng.mg^−1^tissue)	Cytokinin (ng g^−1^DW)	ABA (ng g^−1^DW)	GA (ng g^−1^DW)	IAA (ngmg^−1^tissue)	Cytokinin (ng g^−1^DW)
Rootpac 20/ Dİvadona	Control	170.33f	3.69ab	2.29bc	2.56bc	131.00e	4.44b	3.22bc	3.40bc
	Drought	372.67d	1.51de	1.20 cd	1.75 cd	244.00 cd	3.24c	1.62e	1.97d
	Heat Shock	252.00e	1.96d	1.86c	2.17c	186.67de	2.96 cd	2.24d	2.47 cd
	Drought + Heat Shock	464.00c	1.15e	0.86d	1.16d	291.00c	2.06d	1.22ef	1.48e
Rootpac 40/ Divadona	Control	306.00de	4.44a	3.64a	3.84a	229.33d	6.36a	4.98a	4.97a
	Drought	648.00b	3.07bc	2.66b	2.99b	376.00b	4.06bc	3.08bcd	3.22bc
	Heat Shock	404.33 cd	3.50b	3.02ab	2.88b	311.67bc	4.42b	3.63b	3.76b
	Drought + Heat Shock	939.00a	2.57c	1.95c	1.12d	508.33a	3.33c	2.57 cd	2.86c
Cultivar		**	**	**	**	**	**	**	**
Application		**	**	**	**	**	**	**	**
Cultivar × Application		**	**	**	**	**	**	**	**

Abscisic acid (ABA), Cytokinin, Indole‐3‐acetic acid (IAA), and Gibberellic acid (GA). **Signifcance level: Different letters within the same column signify significant differences between treatments at p ≤ 0.01 (Tukey's HSD test).

#### 3.3.4 Mineral composition

Our findings indicated that the effects of cultivar, treatment, and their interaction on macro mineral content were statistically significant at *p* ≤ 0.01 (Table 3). Nitrogen (N) levels significantly decreased under drought and heat stress conditions. In Rootpac 20/‘Divadona’, N content declined from 3.05% in the control to 1.84% under drought (39.7% decrease) and 2.20% under heat stress (27.9% decrease). The lowest level was observed under combined stress at 1.58%, reflecting a 48.2% reduction. Similarly, in Rootpac 40/‘Divadona’, N levels decreased from 3.69% to 2.06% under drought (44.2% decrease) and 2.55% under heat stress (31.0% decrease), with the lowest value under combined stress (1.73%, 53.1% decrease). Phosphorus (P) content also declined under stress. In Rootpac 20/‘Divadona’, P levels fell from 0.26% in the control to 0.19% under drought (26.9% decrease) and 0.24% under heat stress (7.7% decrease), reaching the lowest level under combined stress (0.11%, 57.7% decrease). A similar trend was observed in Rootpac 40/‘Divadona’, where P content decreased from 0.51% to 0.32% under drought (37.3% decrease) and 0.41% under heat stress (19.6% decrease), with the lowest value under combined stress (0.24%, 52.9% decrease). Potassium (K) levels followed a decreasing trend under stress. In Rootpac 20/‘Divadona’, the control recorded 2.00%, which decreased to 1.38% under drought (31.0% decrease), 1.69% under heat stress (15.5% decrease), and 1.10% under combined stress (45.0% decrease). In Rootpac 40/‘Divadona’, K levels decreased from 2.65% to 1.49% under drought (43.8% decrease) and 1.99% under heat stress (25.0% decrease), with the lowest value under combined stress at 1.31% (50.6% decrease). Calcium (Ca) content exhibited a similar decline. In Rootpac 20/‘Divadona’, Ca levels decreased from 1.63% in the control to 1.15% under drought (29.4% decrease) and 1.55% under heat stress (4.9% decrease), with the lowest level under combined stress (1.03%, 36.8% decrease). In Rootpac 40/‘Divadona’, Ca content decreased from 2.46% to 1.58% under drought (35.8% decrease) and 1.98% under heat stress (19.5% decrease), reaching the lowest value under combined stress at 1.18% (52.0% decline). Magnesium (Mg) content was also significantly affected. In Rootpac 20/‘Divadona’, Mg levels decreased from 0.30% to 0.24% under drought (20.0% decline) and 0.29% under heat stress (3.3% decrease), with the lowest value under combined stress at 0.20% (33.3% reduction). In Rootpac 40/‘Divadona’, Mg content decreased from 0.35% to 0.28% under drought (20.0% decrease) and 0.31% under heat stress (11.4% decrease), with a further decline under combined stress to 0.25% (28.6% decrease). During the recovery phase, mineral contents increased compared to stress conditions but remained below control levels. In Rootpac 20/‘Divadona’, N content rose from 1.58% under combined stress to 1.98%, indicating a 25.3% recovery, while in Rootpac 40/‘Divadona’, N levels increased from 1.73% to 2.16% (24.9% increase). P content in Rootpac 20/‘Divadona’ showed a 90.9% recovery (from 0.11% to 0.21%), while in Rootpac 40/‘Divadona’, it increased by 33.3% (from 0.24% to 0.32%). K, Ca, and Mg contents followed a similar increasing trend, with Rootpac 40/‘Divadona’ showing a 30.5% recovery in K (from 1.31% to 1.71%), a 22.9% increase in Ca (from 1.18% to 1.45%), and a 28.0% improvement in Mg (from 0.25% to 0.32%).

**TABLE 3:** The macro mineral content during stress and recovery in the leaves of the ‘Divadona’ peach cultivar.

	Macro minerals	Stress					Recovery				
Cultivar	Application	N (%)	P (%)	K (%)	Ca (%)	Mg (%)	N (%)	P (%)	K (%)	Ca (%)	Mg (%)
Rootpac 20/ Dİvadona	Control	3.05b	0.26b	2.00b	1.63c	0.30b	3.45b	0.33bc	2.25c	1.85bc	0.37b
	Drought	1.84e	0.19c	1.38 cd	1.15d	0.24c	2.23e	0.28c	1.63e	1.32e	0.28d
	Heat Shock	2.20 cd	0.24bc	1.69bc	1.55 cd	0.29b	2.40d	0.24 cd	1.83d	1.63 cd	0.34bc
	Drought+Heat Shock	1.58f	0.11d	1.10e	1.03e	0.20d	1.98f	0.21d	1.51ef	1.27ef	0.25de
Rootpac 40/ Divadona	Control	3.69a	0.51a	2.65a	2.46a	0.35a	4.61a	0.55a	2.97a	2.92a	0.45a
	Drought	2.06d	0.32b	1.49c	1.58 cd	0.28bc	2.42d	0.40b	1.81d	1.70c	0.35bc
	Heat Shock	2.55c	0.41ab	1.99b	1.98b	0.31ab	2.92c	0.50ab	2.45b	2.25b	0.40ab
	Drought+Heat Shock	1.73ef	0.24bc	1.31d	1.18d	0.25c	2.16ef	0.32bc	1.71de	1.45d	0.32c
Cultivar		**	**	**	**	**	**	**	**	**	**
Application		**	**	**	**	**	**	**	**	**	**
Cultivar × Application		**	**	**	**	**	**	**	**	**	**

Sort alphabetically: Calcium (Ca), Magnesium (Mg), Nitrogen (N), Phosphorus (P), and Potassium (K). **Significance level: Different letters within the same column signify significant differences between treatments at p ≤ 0.01 (Tukey's HSD test).

In the ‘Divadona’ peach cultivar grafted on Rootpac 20/‘Divadona’, stress conditions led to notable decreases in micro mineral content (Table 4). Under drought stress, Mn, Fe, and Zn levels decreased by 12.4%, 13.8%, and 7.9% respectively compared to the control. Heat shock resulted in similar reductions, with Mn decreasing by 10.5%, Fe by 6.7%, and Zn by 5.0%. The combined drought and heat shock treatment showed the most severe reductions, with Mn declining by 23.1%, Fe by 24.7%, and Zn by 10.6% relative to control values. For Rootpac 40/‘Divadona’, the control treatment exhibited higher baseline mineral contents than Rootpac 20/‘Divadona’. Under drought conditions, Mn decreased by 16.9%, Fe by 20.5%, and Zn by 10.7% compared to control. Heat shock stress resulted in less severe reductions, with Mn declining by 7.2%, Fe by 13.2%, and Zn by 8.2%. The combined stressors caused the most pronounced decreases, with Mn reduced by 23.9%, Fe by 27.5%, and Zn by 17.3%. During the recovery phase, mineral contents generally increased across all treatments. In Rootpac 20/‘Divadona’, recovery values for control plants reached 32.31 ppm Mn, 29.95 ppm Fe, and 24.14 ppm Zn. The drought‐stressed plants showed recovery values of 24.93 ppm Mn, 23.96 ppm Fe, and 22.23 ppm Zn. Heat shock treated plants recovered to 27.94 ppm Mn, 25.08 ppm Fe, and 22.73 ppm Zn, while combined stress treatment plants reached 22.07 ppm Mn, 21.04 ppm Fe, and 21.31 ppm Zn. Rootpac 40/‘Divadona’ demonstrated superior recovery capacity, with control plants reaching 39.82 ppm Mn, 32.95 ppm Fe, and 27.82 ppm Zn. Drought‐stressed plants recovered to 32.33 ppm Mn, 25.86 ppm Fe, and 23.92 ppm Zn. Heat shock treated plants showed recovery values of 34.41 ppm Mn, 27.83 ppm Fe, and 24.50 ppm Zn, while combined stress treatment plants recovered to 25.45 ppm Mn, 24.05 ppm Fe, and 22.89 ppm Zn.

**TABLE 4:** The micro mineral content during stress and recovery in the leaves of the ‘Divadona’ peach cultivar.

	Micro Minerals	Stress			Recovery		
Cultivar	Application	Mn (ppm)	Fe (ppm)	Zn (ppm)	Mn (ppm)	Fe (ppm)	Zn (ppm)
Rootpac 20/ Dİvadoa	Control	20.31d	24.79b	20.60c	32.31bc	29.95b	24.14b
	Drought	17.80de	21.37 cd	18.97d	24.93d	23.96d	22.23bc
	Heat Shock	18.18de	23.12bc	19.57 cd	27.94c	25.08c	22.73bc
	Drought+Heat Shock	15.61e	18.66d	18.42d	22.07de	21.04e	21.31c
Rootpac 40/ Divadona	Control	29.38a	30.46a	25.37a	39.82a	32.95a	27.82a
	Drought	24.42c	24.21b	22.66bc	32.33bc	25.86c	23.92b
	Heat Shock	27.27b	26.43ab	23.30b	34.41b	27.83bc	24.50b
	Drought+Heat Shock	22.37 cd	22.08c	20.99c	25.45 cd	24.05 cd	22.89bc
Cultivar		**	**	**	**	**	**
Application		**	**	**	**	**	**
Cultivar × Application		**	**	**	**	**	**

Sort alphabetically: Iron (Fe), Manganese (Mn) and Zinc (Zn).**Significance level: Different letters within the same column signify significant differences between treatments at p ≤ 0.01 (Tukey's HSD test).

### 3.4 General evaluation of morphological, physiological and biochemical responses

**FIGURE 8:**
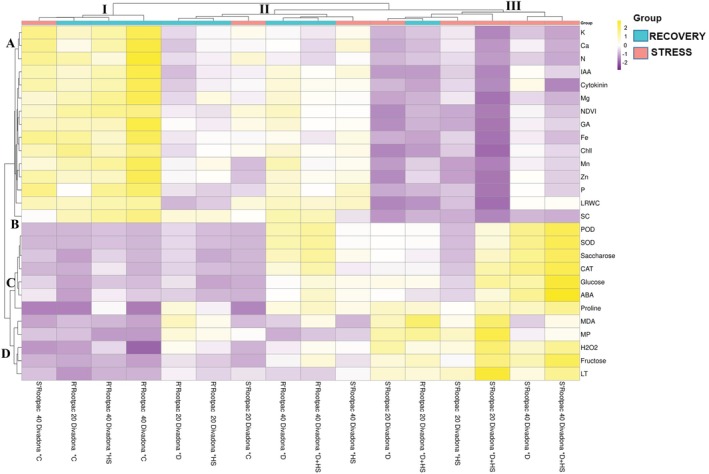
Hierarchical clustering analysis (HCA) for physiological and biochemical parameters in the ‘Divadona’ peach cultivar. Abbreviations: *C (Control), *D (Drought), *D + HS (Drought+Heat Shock), *HS (Heat Shock), ABA (Abscisic acid), Ca (Calcium), CAT (Catalase), Chl (Chlorophyll), Fe (Iron), GA (Gibberellic acid), H₂O₂ (Hydrogen peroxide), IAA (Indole‐3‐acetic acid), K (Potassium), LT (Leaf temperature), LRWC (Leaf relative water content), MDA (Malondialdehyde), Mg (Magnesium), Mn (Manganese), MP (Membrane permeability), N (Nitrogen), NDVI (Normalized Difference Vegetative Index), P (Phosphorus), POD (Peroxidase), R' (Recovery), S′ (Stress), SC (Stomatal Conductance), SOD (Superoxide Dismutase), and Zn (Zinc).

**FIGURE 9:**
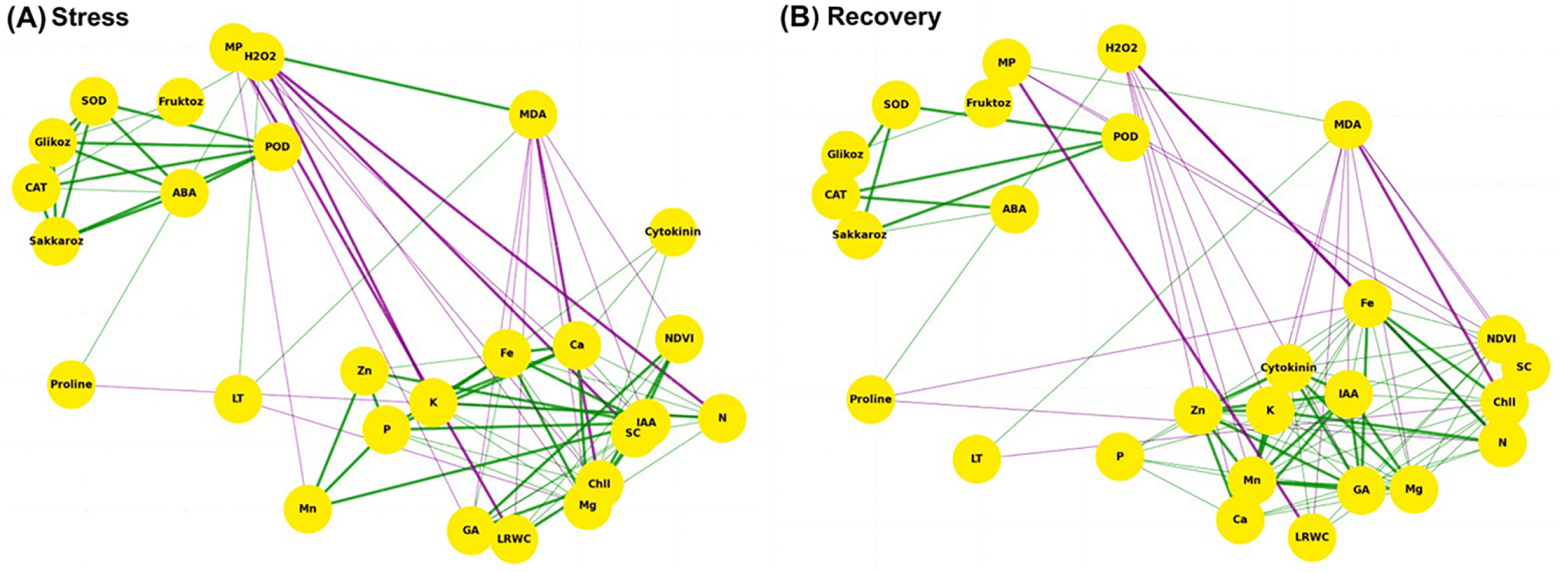
Network Correlation Analysis for physiological and biochemical parameters in the ‘Divadona’ peach cultivar. Abbreviations: ABA (Abscisic acid), Ca (Calcium), CAT (Catalase), Chl (Chlorophyll), Fe (Iron), GA (Gibberellic acid), H₂O₂ (Hydrogen peroxide), IAA (Indole‐3‐acetic acid), K (Potassium), LRWC (Leaf relative water content), LT (Leaf temperature), MDA (Malondialdehyde), Mg (Magnesium), Mn (Manganese), MP (Membrane permeability), N (Nitrogen), NDVI (Normalized Difference Vegetative Index), P (Phosphorus), POD (Peroxidase), SC (Stomatal Conductance), SOD (Superoxide dismutase), Zn (Zinc).

The hierarchical clustering analysis (HCA) revealed the differential responses of various physiological and biochemical parameters in the ‘Divadona’ peach cultivar under stress conditions and during recovery periods (Figure 8). The parameters were organized into four distinct clusters (A‐D) and three major response patterns (I‐III), indicating complex stress‐response mechanisms. Cluster A, which comprised primary nutrients (K, Ca, N), growth regulators (IAA, Cytokinin), photosynthetic parameters (NDVI), and essential minerals (Mg, Fe), exhibited significant reductions under stress conditions. The combined drought and heat shock treatment elicited the most severe decreases in these parameters, while recovery periods demonstrated variable restoration patterns. The antioxidant defense system components (POD, SOD, CAT) and carbohydrate metabolism indicators (Saccharose, Glucose) in Cluster C showed a marked upregulation during stress treatments. This upregulation was particularly pronounced under combined stress conditions, suggesting enhanced oxidative stress responses. Notably, stress markers in Cluster D, including Proline, MDA, MP, and H_2_O_2_, demonstrated substantial accumulation during stress treatments, with the highest levels observed under combined stresses. The clustering patterns indicated that the combined drought and heat shock treatment induced the most severe physiological and biochemical modifications, while the recovery phase showed differential restoration capacities across various parameters. These findings provided comprehensive insights into the stress response mechanisms and recovery dynamics in the ‘Divadona’ peach cultivar under multiple stress conditions. The network correlation analysis revealed distinct relationship patterns among physiological and biochemical parameters during stress and recovery phases in the ‘Divadona’ peach cultivar (Figure 9A‐B). The analysis, based on Pearson correlation coefficients filtered at a threshold of ≥0.9, demonstrated significant parameter interactions. During the stress phase, strong positive correlations (r ≥ 0.95, indicated by thick green lines) were observed between antioxidant enzymes (CAT, SOD, POD) and stress‐related metabolites (Glucose, Fructose, ABA). Notable negative correlations (shown by purple lines) were identified between H_2_O_2_, MP, and MDA with mineral nutrients (Fe, Ca, K) and growth parameters (NDVI, Cytokinin). The stress network structure indicated a complex interplay between oxidative stress markers and essential nutrients. The recovery phase network exhibited reorganized correlation patterns. Strong positive associations emerged among mineral nutrients (N, Mg, Fe) and photosynthetic parameters (Chl, NDVI, SC). The stress markers (H_2_O_2_, MP, MDA) maintained negative correlations with growth‐related parameters but showed reduced connectivity compared to the stress phase. Growth regulators (IAA, GA, Cytokinin) demonstrated enhanced positive correlations with mineral nutrients during recovery.

## 4 DISCUSSION

### 4.1 Effects of drought and heat stress on selected physiological parameters of rootstocks grafted with the ‘Divadona’ peach cultivar

The results of our study demonstrated significant variations in the response of Rootpac 20 and Rootpac 40 rootstocks grafted with the ‘Divadona‘ peach cultivar under drought and heat stress conditions, both individually and in combination. The most notable effects were observed in growth parameters, particularly in shoot development and leaf characteristics, which align with recent findings in the field (Bolat et al., 2024). One of the most striking observations in our study was the superior performance of Rootpac 40/‘Divadona’ compared to Rootpac 20/‘Divadona’ across all measured parameters under stress conditions. For instance, Rootpac 40/‘Divadona’ maintained higher relative shoot diameter (RSD) values under combined stress (19.75%) compared to Rootpac 20/‘Divadona’ (14.99%). This differential response suggests a more robust stress tolerance mechanism in Rootpac 40/‘Divadona’, which is consistent with previous studies on *Prunus* rootstocks (Jiménez et al., 2013; Iacona et al., 2019). The maintenance of higher leaf area in Rootpac 40/‘Divadona’ under combined stress conditions (11.6% decrease vs. control) compared to Rootpac 20/‘Divadona’ (15.4% decrease) was particularly noteworthy. This ability to maintain photosynthetic capacity under stress conditions could be a crucial factor in the rootstock's superior performance. As suggested by Opazo et al. (2020) and Balfagón et al. (2022), the interaction between rootstock characteristics and scion performance plays a vital role in determining overall stress tolerance. The root system responses observed in our study provided interesting insights into adaptation strategies. While both rootstocks showed reduced root length under drought stress, Rootpac 20/‘Divadona’ exhibited an unexpected 4.0% increase in root length under heat stress, whereas Rootpac 40/‘Divadona’ showed a slight decrease (1.6%). This differential response could be interpreted as an adaptation strategy, similar to what Farnia et al. (2015) described as roots seeking deeper water sources under stress conditions. The combined stress treatment resulted in the most severe impacts on growth parameters, supporting the concept of synergistic effects between multiple stress factors (Suzuki et al., 2014). This was particularly evident in the fresh and dry weight measurements, where the combined stress led to more substantial reductions than either stress alone. For instance, the fresh shoot weight in Rootpac 20/‘Divadona’ decreased by 19.2% under combined stress, compared to 9.9% and 8.4% under individual drought and heat stress, respectively. The hierarchical clustering analysis of morphological parameters revealed distinct stress response patterns and recovery mechanisms, providing novel insights into plant adaptation under multiple stress conditions. Our findings demonstrated the complex interplay between various cellular processes and stress responses, highlighting both specific and shared adaptation mechanisms. The observed clustering of growth‐related parameters and their significant reduction under stress conditions, particularly in combined stress treatments, aligns with previous findings by You et al. (2019), who reported similar growth inhibition patterns under drought stress. The strong negative correlations between growth parameters and stress markers (H₂O₂, MP, MDA) suggest an adaptive response mechanism whereby plants redirect resources from growth to stress tolerance, consistent with the growth‐defense trade‐off model proposed by Jiménez et al. (2013). Notably, the variable restoration patterns observed during the recovery phase, characterized by enhanced positive correlations with mineral nutrients, indicate a sophisticated recovery mechanism that may be modulated by nutrient availability and allocation, supporting earlier observations by Ben Yahmed et al. (2020).

### 4.2 Some physiological responses of the ‘Divadona’ peach cultivar to stress and recovery

The superior performance of the ‘Divadona‘ peach cultivar when grafted on Rootpac 40/‘Divadona’ suggests an effective transfer of stress tolerance characteristics from rootstock to scion. This interaction between rootstock and scion involves complex biological processes, including cell recognition, cell cycle regulation, and plasmodesmata formation at the graft union (Pina et al., 2012). The success of this interaction is crucial for developing stress‐resistant grafted combinations. The consistent underperformance of Rootpac 20/‘Divadona’ across all parameters aligns with previous research indicating its lower vigor levels compared to other rootstocks (Jiménez et al., 2013; Mestre et al., 2017). This pattern suggests that rootstock selection should carefully consider the intended growing environment, particularly in regions prone to multiple environmental stresses. An interesting aspect of our findings is the differential impact of stress treatments on below‐ground versus above‐ground biomass. The relatively smaller reduction in root parameters compared to shoot parameters under stress conditions suggests a possible resource allocation strategy favoring root system maintenance. This adaptation could be crucial for survival under stress conditions, as maintaining root system functionality is essential for water and nutrient uptake. Our results, combined with existing literature, emphasize the importance of rootstock selection in determining the success of grafted peach trees under stress conditions. The ability of certain rootstocks to maintain growth and physiological stability under stress conditions can significantly influence the performance of the grafted scion, as demonstrated by the superior performance of the Rootpac 40/‘Divadona’ combination in our study.

The present study showed that the physiological responses of ‘the ‘Divadona” peach cultivar to various stress conditions revealed insights into stress tolerance mechanisms and recovery patterns. Our findings demonstrated that stomatal conductance exhibited substantial reductions under stress conditions, with the most severe decline (62.7%) observed under combined drought and heat stress (Figure 2A‐B). This response aligns with previous research indicating that stomatal closure is a primary physiological response to water deficiency (Chaves et al., 2003). The differential responses observed between rootstocks, particularly Rootpac 40/‘Divadona”s superior ability to maintain higher stomatal conductance under stress conditions, suggest the presence of more efficient water management strategies, possibly attributed to enhanced root system architecture or osmotic adjustment mechanisms (Jiménez et al., 2013). Regarding analysis of leaf relative water content (LRWC), the observed 34.9% reduction in LRWC under combined stress (Figure 3A‐B) indicates the severity of multiple stress factors on plant water relations. This finding is particularly significant as it demonstrates the synergistic effect of combined stresses, which has been increasingly recognized in plant stress physiology (Suzuki et al., 2014). The superior performance of Rootpac 40/‘Divadona’ in maintaining higher LRWC values suggests enhanced stress tolerance mechanisms, potentially through better water use efficiency or root system development. In our results, membrane permeability showed significant increases under stress conditions, with the most pronounced effect (92.6% increase) observed under combined stress (Figure 2E‐F). This substantial increase in membrane permeability, particularly evident in Rootpac 20/‘Divadona’ combinations, aligns with previous studies showing that abiotic stresses can damage cell membranes, leading to increased electrolyte leakage (Demidchik et al., 2014). The observed changes likely result from reactive oxygen species (ROS) accumulation, leading to lipid peroxidation and protein denaturation (Sharma et al., 2012). The more severe impact of combined stresses on membrane integrity emphasizes the complex nature of multiple stress responses, a phenomenon gaining increasing attention in plant stress physiology (Zandalinas et al., 2018).

Chlorophyll content variations under stress conditions provided valuable insights into photosynthetic capacity and leaf health. The observed 27.6% reduction in chlorophyll content under combined stress (Figure 2C‐D) suggests a significant impairment of the photosynthetic machinery. This finding corresponds with previous research indicating that oxidative stress and accelerated leaf senescence can contribute to chlorophyll degradation under stress conditions (Farooq et al., 2009). The differential responses between rootstocks in maintaining chlorophyll content suggest varying levels of stress tolerance mechanisms. Thermal imaging analysis (Figure 4A‐D) revealed distinct temperature distribution patterns among rootstocks, with Rootpac 40/‘Divadona’ maintaining lower leaf temperatures under stress conditions. This superior thermal regulation ability suggests more efficient water use or better‐developed root systems (Grossiord et al., 2020). The observed increases in leaf temperature, particularly in Rootpac 20/‘Divadona’ under combined stress, indicate compromised transpirational cooling, likely due to stomatal closure in response to water deficit (Urban et al., 2017). These findings align with previous studies documenting the impact of abiotic stresses on plant leaf temperature (Shafqat et al., 2021). The recovery patterns observed post‐stress period provide valuable insights into stress memory and adaptation mechanisms. While some physiological parameters showed partial recovery, the persistent effects, particularly in Rootpac 20/‘Divadona’, indicate potential lasting damage to plant functions, a phenomenon known as stress priming (Crisp et al., 2016). The superior recovery capability of Rootpac 40/‘Divadona’ in terms of both stomatal conductance and LRWC suggests more robust stress mitigation mechanisms, making it potentially more suitable for regions with limited water resources or increasing drought and temperature stress conditions due to climate change. The NDVI results further support these findings, with significant reductions observed under stress conditions, particularly in Rootpac 20/‘Divadona’ combinations. These decreases may be attributed to reduced photosynthetic activity and overall plant health deterioration (Guo et al., 2016). Additionally, the relationship between NDVI values and nutrient content, particularly nitrogen, has been previously documented (Dogan et al., 2025), suggesting potential interactions between stress responses and nutrient utilization efficiency. On the other hand, the hierarchical clustering analysis of physiological parameters revealed distinct stress response patterns and recovery mechanisms, providing novel insights into plant adaptation under multiple stress conditions (Figure 8). The distinct clustering of photosynthetic parameters within Cluster A and their significant reduction under combined drought and heat stress reveals the vulnerability of photosynthetic machinery to multiple stresses. These findings extend previous work by Chen et al., (2021), who documented similar photosynthetic inhibition under individual stress conditions. The strong positive correlations between physiological indicators and mineral nutrients during recovery suggest that nutrient homeostasis plays a crucial role in photosynthetic recovery, a phenomenon not previously emphasized in stress response studies. This relationship provides new insights into the mechanistic basis of stress recovery and suggests potential intervention strategies for enhancing stress resilience.

### 4.3 Biochemical responses of the ‘Divadona’ peach cultivar to stress and recovery

The findings of our study provided a comprehensive understanding of the biochemical and physiological responses of the “Divadona” peach cultivar grafted on Rootpac 20 and Rootpac 40 rootstocks under drought, heat, and combined stress conditions. The results highlighted significant changes in oxidative stress markers, osmoregulation, antioxidant enzyme activities, hormonal balance, and mineral nutrient content, which are critical for understanding the stress tolerance mechanisms in peach plants. Regarding oxidative stress and antioxidant responses, the observed increase in H₂O₂ levels under combined drought and heat stress, particularly in Rootpac 20/‘Divadona’ (Figure 5E‐F), is consistent with previous studies indicating that abiotic stresses enhance ROS production in plants (Mittler et al., 2022). The elevated H₂O₂ levels suggest cellular homeostasis disruption and increased metabolic imbalances under stress conditions (Noctor et al., 2014). This oxidative stress can impair cellular defense mechanisms and threaten long‐term plant health (Dogan et al., 2025). The higher MDA accumulation in Rootpac 20/‘Divadona’ under combined stress (Figure 5A‐B) further supports the notion that drought and heat stress compromise membrane integrity and increase cellular damage (Hussain et al., 2018). These findings align with the hypothesis that oxidative stress is a key factor in stress‐induced damage in plants. In contrast, Rootpac 40/‘Divadona’ exhibited lower MDA accumulation and higher proline content under stress (Figure 5C‐D), suggesting superior stress tolerance. Proline, a well‐known osmoprotectant, helps plants mitigate water loss and maintain osmotic balance under stress (Szabados and Savouré, 2010). The significant proline accumulation in Rootpac 40/‘Divadona’ indicates enhanced osmoregulation mechanisms, which may contribute to its higher stress tolerance. This is further supported by the sustained high activity of antioxidant enzymes such as catalase (CAT), superoxide dismutase (SOD), and peroxidase (POD) in Rootpac 40/‘Divadona’ during stress and recovery (Figure 6A‐C). These enzymes play a crucial role in scavenging ROS and mitigating oxidative damage (Gill & Tuteja, 2010). The persistent elevation of antioxidant enzyme activities in Rootpac 40/‘Divadona’, even during recovery, suggests a robust stress memory mechanism, which may enhance future stress responses (Bruce et al., 2007; Dogan et al., 2025).

The hormonal analysis revealed a significant increase in abscisic acid (ABA) levels under stress, particularly in Rootpac 40/‘Divadona’ (Table 2). ABA is a key hormone in stress signaling, regulating stomatal closure to reduce water loss and activating adaptive mechanisms (Balfagón et al., 2022). The higher ABA accumulation in Rootpac 40/‘Divadona’ under combined stress (939.00 ng g^−1^ D.W) compared to Rootpac 20/‘Divadona’ (464.00 ng g^−1^ D.W) suggests a more efficient stress response in the former. Conversely, the decline in gibberellic acid (GA), indole‐3‐acetic acid (IAA), and cytokinin levels under stress indicates a trade‐off between growth and stress adaptation. The partial recovery of these hormones during the recovery phase highlights the dynamic nature of hormonal regulation in response to stress. Considering mineral nutrient dynamics, the reduction in macro‐ and micronutrient content under stress conditions (Table 3 and Table 4) is consistent with previous studies showing that abiotic stresses impair nutrient uptake and translocation (Ahanger et al., 2016). The significant decline in nitrogen (N), phosphorus (P), potassium (K), calcium (Ca), and magnesium (Mg) levels under combined stress in Rootpac 20/‘Divadona’ (Figure 7A‐C) suggests that nutrient metabolism is highly sensitive to drought and heat stress. This is particularly evident in the severe reduction of N content (48.2%) and P content (57.7%) under combined stress in Rootpac 20/‘Divadona’. The lower nutrient uptake may be attributed to reduced root activity and impaired membrane function under stress (Hu and Schmidhalter, 2005).

In contrast, Rootpac 40/‘Divadona’ maintained higher nutrient levels under stress and showed better recovery, indicating superior nutrient uptake and translocation mechanisms. The higher Ca and Mg retention in Rootpac 40/‘Divadona’ (Table 3) may contribute to its enhanced stress tolerance, as these nutrients are critical for maintaining cell wall integrity and chlorophyll synthesis (Cakmak & Kirkby, 2008). The better recovery of micronutrients such as manganese (Mn), iron (Fe), and zinc (Zn) in Rootpac 40/‘Divadona’ (Table 4) further supports its superior stress adaptation capacity. These findings suggest that genotypic differences in nutrient use efficiency play a significant role in stress tolerance (Sardans and Peñuelas, 2012). On the other side, the hierarchical clustering analysis of biochemical parameters revealed distinct stress response patterns and recovery mechanisms, providing novel insights into plant adaptation under multiple stress conditions. The dynamic responses observed in biochemical parameters, particularly the formation of distinct clusters (Figure 8C‐D), demonstrate the complexity of cellular stress responses. The marked upregulation of antioxidant enzymes (POD, SOD, CAT) under combined stress conditions extends previous findings by Zhang et al. (2024), who reported similar antioxidant responses under single stress conditions. The strong positive correlations among antioxidant enzymes and stress‐related metabolites during the stress phase, followed by reduced connectivity during recovery, suggest a coordinated but flexible biochemical response system. This pattern aligns with the “stress response flexibility” hypothesis proposed by Gill et al. (2016), while adding new insights into recovery dynamics. The enhanced positive correlations between growth regulators and mineral nutrients during recovery represent a novel finding that may have significant implications for understanding stress adaptation mechanisms. This relationship suggests that nutrient status may play a more central role in stress recovery than previously recognized, potentially through the modulation of hormone signaling pathways (Jurado‐Mañogil et al., 2024).

## 5 CONCLUSION

Our results revealed that the ‘Divadona’ peach cultivar exhibited distinct physiological and biochemical responses when grafted onto Rootpac 20 and Rootpac 40 rootstocks under drought stress, heat shock, and their combination. The investigation demonstrated that combined stress treatments induced the most severe modifications across all measured parameters, with Rootpac 40/‘Divadona’ consistently displaying superior stress tolerance compared to Rootpac 20/‘Divadona’. This enhanced tolerance was evidenced by better maintenance of growth parameters, including relative shoot diameter and relative shoot length under control conditions. Hierarchical clustering analysis identified four distinct response clusters, highlighting the coordinated upregulation of antioxidant defense systems (POD, SOD, CAT) alongside increased stress markers (Proline, MDA, H₂O₂). Network correlation analysis confirmed strong positive correlations between antioxidant enzymes and stress‐related metabolites, while revealing negative correlations between oxidative stress markers and essential nutrients. During the recovery phase, both rootstocks exhibited differential recovery capacities, with Rootpac 40 demonstrating superior recovery potential, particularly in mineral nutrient content and photosynthetic parameters. The reorganization of correlation patterns during recovery, especially between growth regulators and mineral nutrients, suggested active physiological adjustment mechanisms. In conclusion, this study provided valuable insights into the biochemical and physiological mechanisms underlying stress tolerance in the ‘Divadona’ peach cultivar. The findings underscore the importance of selecting rootstocks with robust stress adaptation mechanisms to improve crop resilience under changing climatic conditions. Future research should focus on elucidating the genetic and molecular basis of these stress tolerance traits to facilitate the development of stress‐resistant peach varieties.

## AUTHOR CONTRIBUTIONS

I.B. and M.D. contributed to the investigation, methodology, and conceptualization of the study. I.B., M.D., O.K., and M.T. were responsible for the draft preparation, visualization, software, and formal analysis. I.B. and O.K. contributed to the writing‐original and writing‐review and editing. All authors have read and approved the final manuscript.

## CONFLICT OF INTEREST STATEMENT

The authors declare no known competing financial interests or personal relationships that could have influenced the work reported in this scientific article.

## Data Availability

The data supporting the findings of this study are available from the corresponding author upon reasonable request.
